# Multi‐proteomic analyses of 5xFAD mice reveal new molecular signatures of early‐stage Alzheimer's disease

**DOI:** 10.1111/acel.14137

**Published:** 2024-03-04

**Authors:** Seulah Lee, Kuk‐In Jang, Hagyeong Lee, Yeon Suk Jo, Dayoung Kwon, Geuna Park, Sungwon Bae, Yang Woo Kwon, Jin‐Hyeok Jang, Yong‐Seok Oh, Chany Lee, Jong Hyuk Yoon

**Affiliations:** ^1^ Neurodegenerative Diseases Research Group Korea Brain Research Institute Daegu Republic of Korea; ^2^ Cognitive Science Research Group Korea Brain Research Institute Daegu Republic of Korea; ^3^ Department of Brain‐Cognitive Science Daegu‐Gyeongbuk Institute of Science and Technology (DGIST) Daegu Republic of Korea

**Keywords:** Alzheimer's disease, biomarker, early‐stage Alzheimer's disease, extracellular vesicle, machine learning, proteomics

## Abstract

An early diagnosis of Alzheimer's disease is crucial as treatment efficacy is limited to the early stages. However, the current diagnostic methods are limited to mid or later stages of disease development owing to the limitations of clinical examinations and amyloid plaque imaging. Therefore, this study aimed to identify molecular signatures including blood plasma extracellular vesicle biomarker proteins associated with Alzheimer's disease to aid early‐stage diagnosis. The hippocampus, cortex, and blood plasma extracellular vesicles of 3‐ and 6‐month‐old 5xFAD mice were analyzed using quantitative proteomics. Subsequent bioinformatics and biochemical analyses were performed to compare the molecular signatures between wild type and 5xFAD mice across different brain regions and age groups to elucidate disease pathology. There was a unique signature of significantly altered proteins in the hippocampal and cortical proteomes of 3‐ and 6‐month‐old mice. The plasma extracellular vesicle proteomes exhibited distinct informatic features compared with the other proteomes. Furthermore, the regulation of several canonical pathways (including phosphatidylinositol 3‐kinase/protein kinase B signaling) differed between the hippocampus and cortex. Twelve potential biomarkers for the detection of early‐stage Alzheimer's disease were identified and validated using plasma extracellular vesicles from stage‐divided patients. Finally, integrin α‐IIb, creatine kinase M‐type, filamin C, glutamine γ‐glutamyltransferase 2, and lysosomal α‐mannosidase were selected as distinguishing biomarkers for healthy individuals and early‐stage Alzheimer's disease patients using machine learning modeling with approximately 79% accuracy. Our study identified novel early‐stage molecular signatures associated with the progression of Alzheimer's disease, thereby providing novel insights into its pathogenesis.

## Graphical abstract

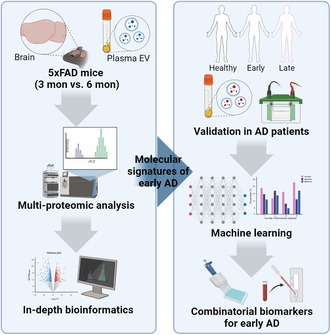

**Abbreviations**
A2M: alpha‐2 macroglobulin
AD: Alzheimer's disease
Akt: protein kinase B
ALIX: ALG‐2‐interacting protein X
ANOVA: analysis of variance
APP: amyloid precursor protein
Atp4a: ATPase H^+^/K^+^ transporting subunit alpha
AUC: area under the curve
BP: biological processes
Cadps2: calcium dependent secretion activator 2
Capzb: F‐actin capping protein subunit beta
CC: cellular components
CD: cluster of differentiation
CK: creatine kinase
CKM: creatine kinase M‐type
CSF: cerebrospinal fluid
CT: computed tomography
EDTA: ethylenediaminetetraacetic acid
EIF2: eukaryotic initiation factor 2
ER: endoplasmic reticulum
EV: extracellular vesicles
GO: gene ontology
Hp: haptoglobin
HPC: hippocampus
HRP: horseradish peroxidase
HSP: heat shock protein
HSPA1L: heat shock 70 kDa protein 1‐like
IPA: ingenuity pathway analysis
ITGA2B: integrin alpha‐IIb
LXR/RXR: liver X receptor/retinoid X receptor
MAN2B1: lysosomal alpha‐mannosidase
MCI: mild cognitive impairment
MF: molecular functions
ML: machine learning
MMSE: mini‐mental state examination
mPFC: medial prefrontal cortex
MRI: magnetic resonance imaging
ORM2: orosomucoid 2
PAGE: polyacrylamide gel electrophoresis
PBS: phosphate‐buffered saline
PF4: platelet factor 4
PFA: paraformaldehyde
PI3K: phosphoinositide 3‐kinase
PLTP: phospholipid transfer protein
PSM: peptide spectral match
PVDF: polyvinylidene difluoride
QSOX1: sulfhydryl oxidase 1
RIPA: radioimmunoprecipitation assay
ROC: receiver operating characteristic
RT: room temperature
SDS: sodium dodecyl sulphate
SE: standard error
SEM: standard error of the mean
SEN: sensitivity
Sgip1: SH3GL interacting endocytic adaptor 1
Slc25a31: solute carrier family 25 member 31
SPE: specificity
SVM: support vector machine
TGM2: protein‐glutamine gamma‐glutamyltransferase 2
TLN: talin
Tpp2: tripeptidyl peptidase 2
tuba3b: tubulin alpha 3b
VDAC: voltage‐dependent anion‐selective channel protein
WB: western blotting
WT: wild‐type

## 1 INTRODUCTION

Alzheimer's disease (AD) is the most common type of dementia worldwide and is associated with memory deficits and cognitive decline. It is primarily characterized by amyloid beta (Aβ) plaque deposition and neurofibrillary tangles of hyperphosphorylated tau (Weller & Budson, 2018). The exact etiology of AD has not been elucidated, similar to most neurodegenerative diseases; however, a characteristic and substantial reduction in cholinergic neurons in the forebrain has been reported (Ferreira‐Vieira et al., 2016). The current treatment for AD includes the use of acetylcholinesterase inhibitors and *N*‐methyl‐d‐aspartate receptor antagonists (Hughes et al., 2016; Masters et al., 2015); however, these treatments are only effective in early‐stage AD, as their efficacy in late‐stage AD remains poor (Cummings et al., 2022; Wattmo & Wallin, 2017). Therefore, an early diagnosis and rapid therapy can effectively delay AD progression.

Various diagnostic tools that involve the use of blood and cerebrospinal fluid (CSF) samples have been developed for AD (O'Bryant, 2016). CSF collection is invasive and inconvenient, as it can only be obtained through lumbar puncture (O'Bryant et al., 2017). In addition, although brain imaging, such as amyloid‐positron emission tomography and magnetic resonance imaging, can help detect brain atrophy, brain atrophy is only evident when it is quite advanced. Moreover, it is expensive and difficult to apply these modalities in clinical settings owing to time‐consuming data analysis and the requirement for specialized personnel (Kim et al., 2022). Instead, analysis of blood‐based biomarker is minimally invasive, more convenient, and cost‐ and time‐efficient compared to CSF sampling or brain imaging (O'Bryant et al., 2017). Therefore, blood‐based biomarkers can practically be applied at the population level, and previous research has focused on identifying blood‐based biomarkers for AD diagnosis (Laske et al., 2011). However, attempts to cross‐validate these methods have been unsuccessful (Karikari et al., 2022; O'Bryant et al., 2017). Systematic omics have been used to evaluate apolipoprotein J, sphingolipids, ceramides, monohexosylceramides, and phospholipids as candidate biomarkers of AD (Byeon et al., 2021; Dinkins et al., 2017; O'Bryant, 2016); however, no reliable molecular targets have been identified in the blood for AD diagnosis.

Extracellular vesicles (EVs) are abundant secretory components in the blood and mediate signaling that transports intracellular proteins, nucleic acids, and lipids in response to para‐endocrine and endocrine signals (Beer & Wehman, 2017; Mentkowski et al., 2018; Wahlund et al., 2017). Moreover, EVs are resistant to enzymes and phagocytosis by immune cells, and they can even penetrate the blood–brain barrier bidirectionally (Zhang et al., 2020). Based on these characteristics, an increasing number of studies have explored the potential utility of EV markers in predicting and detecting AD and other neurodegenerative diseases (Ciregia et al., 2017; Croese & Furlan, 2018). The efficacy of neuronally derived EVs has been used to predict the progression from mild cognitive impairment (MCI) to dementia (Winston et al., 2016). Individuals who progressed from MCI to AD within 36 months exhibited alterations in the plasma levels of neuronally derived EVs containing p‐tau, Aβ_1‐42_, neurogranin, and repressor element 1‐silencing transcription factor, compared with healthy individuals and those with stable MCI (Winston et al., 2016). However, further validation is necessary to determine the accuracy of available data regarding EV‐derived biomarkers (Aharon et al., 2020). Additionally, there is a lack of methodological standardization of reliable, systematic proteomic blood‐based EV profiling.

Many types of familial AD mouse models have been developed, most of which have mutations in *APP* or *PSEN1* or both, but their characteristics are slightly different (Esquerda‐Canals et al., 2017). AD models such as Tg2576, PDAPP, APP23, and J20 manifest Aβ pathology later than cognitive impairment. Instead, the TgCRND8, PS2APP, and Tg‐ArcSwe models manifest both Aβ pathology and cognitive impairment almost simultaneously (Jankowsky & Zheng, 2017; Li et al., 2011). In the case of 5xFAD mice, it has been reported that Aβ accumulation starts at around 2 months (Youmans et al., 2012), but cognitive impairment is observed from ~5–6 months onwards (Smith & Hopp, 2023; Wei et al., 2016). Recently, pathophysiological changes including accumulation of Aβ and tau have been detected many years before the clinical manifestation of AD (Aisen et al., 2017; Palmqvist et al., 2017). Based on previous reports, we aimed to find molecular signatures potentially associated with early AD in 5xFAD mice and to evaluate their diagnostic potential in AD patients.

Therefore, we performed reliable comparative proteomic analyses of the multi‐proteomes in the cortex, hippocampus, and plasma EV of 3‐ and 6‐month‐old 5xFAD mice. Furthermore, we used a machine learning (ML) model to validate the accuracy of the identified biomarkers in distinguishing healthy individuals from those in the early stages of AD. We believe that our findings will facilitate the identification and validation of the clinical potential of candidate biomarkers and will provide new insights into understanding AD pathogenesis.

## 2 METHODS

### 2.1 Experimental animals and sample preparations

All experimental procedures involving animals were approved by the Animal Use and Care Committee of the Korea Brain Research Institute (approval number IACUC‐20‐00038). 5xFAD hemizygous (B6.Cg‐Tg [APPSwFlLon, PSEN1*M146L*L286V] 6799Vas/Mmjax), MMRRC stock (#34848), and wild type (WT) littermates were produced by mating with C57BL/6J (JAX stock #000664) females (Jackson Laboratory, Bar Harbor, ME, USA). The mice were maintained under a 12 h light/dark cycle with ad libitum access to food and water. Male and female littermates aged 3 and 6 months were used in this study.

Histological studies involved anesthetizing the mice with carbon dioxide and intracardially perfusing with 0.9% normal saline. The mice were fixed in a solution containing 4% paraformaldehyde (PFA) in 0.1 M phosphate‐buffered saline (PBS); next, their brains were removed, placed in the same fixative solution at 4°C overnight, and then transferred to a 30% (w/v) sucrose solution. Cryoprotected brains were serially sliced into 40 μm sections in the coronal plane using a cryostat (CM1950; Leica, Wetzlar, Germany) and stored at 4°C in Dulbecco's PBS (DPBS) solution containing 0.1% sodium azide.

For biochemical and proteomic analyses, the mice were anesthetized with carbon dioxide and intracardially perfused with 0.9% normal saline. Their brains were removed, washed with ice‐cold PBS, dissected into the cortex and hippocampus for analyses, immediately snap‐frozen, and stored at −80°C until further use. Blood samples were extracted before cardiac perfusion. Approximately, 500 μL of whole blood was transferred to an ethylenediaminetetraacetic acid (EDTA)‐coated container (BD, NJ, USA) and centrifuged at 3000× g for 15 min at 4°C to separate the plasma.

### 2.2 Aβ staining

Brain sections were stained with 0.5% Thioflavin‐S solution (Sigma‐Aldrich, St. Louis, MO, USA) for 10 min at room temperature (RT; 25 ± 2°C), then blocked in 20 mM Tris‐buffered saline/0.1% Triton X‐100/3% goat serum (TBS‐TS) for 30 min at RT, and incubated with the primary antibody (anti‐6E10; mouse monoclonal; BioLegend (SIG‐39320), San Diego, CA, USA) in TBS‐TS overnight at 4°C. The sections were then washed in TBS, incubated with secondary anti‐mouse IgG labeled with Alexa Fluor 568 for 3 h at RT, rewashed with TBS, and mounted onto slides with VECTASHIELD® Antifade Mounting Medium containing 4′,6‐diamidino‐2‐phenylindole (DAPI) (Vector Laboratories, Newark, CA, USA). Images were acquired using a panoramic scanning system (3DHistech, Budapest, Hungary).

### 2.3 Peptide generation through in‐solution brain tissue digestion

Peptides were prepared from the hippocampal and cortical tissues of 3‐ and 6‐month‐old 5xFAD mice using customized in‐solution digestion. Briefly, the hippocampus and cortex of 3‐ and 6‐month‐old 5xFAD mouse brains were dissected and washed with PBS. Each tissue was dissolved in lysis buffer (40 mM ammonium bicarbonate, pH 7.8) supplemented with 1% ProteaseMAX (Promega, Madison, WI, USA), sonicated (30% amplitude for 3 s on and 10 s off, 10 times), and incubated on ice for 30 min. The lysate was diluted 4× with 40 mM ammonium bicarbonate buffer, incubated with 10 mM dithiothreitol (Sigma‐Aldrich) at 56°C for 20 min, and treated with 20 mM iodoacetamide (Sigma‐Aldrich) at RT for 20 min in the dark. Protein concentrations were quantified using the bicinchoninic acid protein assay reagent (Thermo Fisher Scientific, Waltham, MA, USA), and 100 μg of the protein was used for subsequent processing. The samples were treated with trypsin‐Lys C mixture (Promega) at a 1:50 ratio for 4 h at 50°C. The reaction was quenched by adding 0.5% trifluoroacetic acid. The trypsin‐digested peptides were lyophilized and desalted using a desalting column (#89873, Thermo Fisher Scientific) according to the manufacturer's protocol.

### 2.4 EV preparation

Whole‐blood samples were collected for plasma preparation using EDTA treatment and centrifugation. Plasma EVs were prepared using a stepwise ultracentrifugation protocol according to existing methods with some modifications. Briefly, plasma samples were diluted 10× using PBS, incubated for 60 min at 4°C, and centrifuged at 12,000× g for 20 min at 4°C in a tabletop centrifuge (Eppendorf, Hamburg, Germany). The pellets were resuspended in 1 mL PBS and centrifuged twice at 120,000× g for 90 min at 4°C. The precipitated pellets were resuspended in 200 μL PBS, and the EV protein concentration was determined using a bicinchoninic acid protein assay. The size of EVs was estimated using NanoSight LM10 (Malvern Panalytical, Malvern, UK) according to the manufacturer's instructions. Peptide preparation involved resuspending the EV samples in lysis buffer containing 1% ProteaseMAX and 40 mM ammonium bicarbonate (pH 7.8) and processing as described above.

### 2.5 Mass analysis and database search

The digested peptides were analyzed using a liquid chromatography–tandem mass spectrometry system (LC–MS/MS) consisting of an UltiMate™ 3000 RSLCnano system (Thermo Fisher Scientific) and an Orbitrap Eclipse Tribrid mass spectrometer (Thermo Fisher Scientific) equipped with a nanoelectrospray source (EASY‐Spray Sources, Thermo Fisher Scientific). The peptides were bound to a 75 μm × 2 cm C18 pre‐column (nanoViper, Acclaim PepMap100, Thermo Fisher Scientific), followed by separation on an analytical C18 column (75 μm × 50 cm PepMap RSLC, Thermo Fisher Scientific). The peptides were separated using a 140 min discontinuous gradient of 5%–25% acetonitrile and 0.1% formic acid at a flow rate of 250 nL/min and an electrospray voltage set at 2000 V. The mass spectrometer was operated in a data‐dependent mode that automatically switched between MS1 and MS2 during chromatographic separation. Mass spectrometer calibration was performed using the proposed calibration solution according to the manufacturer's instructions. Mass spectrometry data were acquired using the following parameters: full‐scan MS1 spectra (400–1600 *m/z*) were acquired at a resolution of 60,000 and an automatic gain control target value of 4.0e5 for a maximum ion injection time of 100 ms. MS2 spectra were acquired using a mass analyzer at a resolution of 60,000 with high‐energy collision dissociation of 30% normalized collision energy, an automatic gain control target value of 1.0e5, and a maximum ion injection time of 300 ms. Previously fragmented ions were excluded for 20 s.

Tandem mass spectral data were processed using Thermo Fisher Scientific Proteome Discoverer version 2.41 (Thermo Fisher Scientific). Spectral data were searched against the mouse UniProt database (release version 2020_09). The analysis workflow included four nodes: Spectrum Files (data input), Spectrum Selector (spectrum and feature retrieval), Sequest HT (sequence database search), and Percolator (peptide spectral match [PSM] or PSM validation and false discovery rate analysis). All identified proteins had a false discovery rate of ≤1% calculated at the peptide level. Validation was based on the *q*‐value. Search parameters allowed for tryptic specificity of up to two missed cleavages, with methylthio‐modifications of cysteine as a fixed modification and methionine oxidation as a dynamic modification. The mass search parameters for +1, +2, and +3 ions included mass error tolerances of 20 ppm and 0.6 Da for precursor and fragment ions, respectively.

A normalized peptide spectrum match index was applied to calculate quantitative changes in the identified proteins among the experimental groups. The peptide spectrum match index was calculated for each protein; this is the cumulative peptide spectrum match from each technical replicate. The *G*‐test was performed for peptide spectrum matches to estimate the statistical confidence for fold changes in identified proteins between experimental groups (Astarita et al., 2018; Hayashi et al., 2015; Kim et al., 2010; Nestler et al., 2012).

### 2.6 Bioinformatics analysis

The Database for Annotation, Visualization, and Integrated Discovery Bioinformatics Resources v. 6.8 and ingenuity pathway analysis (IPA) were used for Gene Ontology (GO)‐based functional annotations and in‐depth bioinformatics analysis, respectively. UniProt protein accession numbers of the identified proteins were coupled with the normalized fold changes between WT and 5xFAD and uploaded to the IPA using the protein expression criteria. The following criteria were used for quantitative pathway analysis: *Z*‐score cut‐off = 0.5, −log (*p*‐value) >1.3.

### 2.7 Immunoblotting

Western blot analysis was performed to semi‐quantitatively determine the levels of the proteins of interest. The total protein was extracted using radioimmunoprecipitation assay buffer containing 1× Halt protease and a phosphatase inhibitor cocktail (Thermo Fisher Scientific). The protein concentration was measured using the bicinchoninic acid protein assay (Thermo Fisher Scientific). Protein samples were mixed with the sodium dodecyl sulfate sample buffer (Bio‐Rad) containing 10% beta‐mercaptoethanol and incubated for 5 min at 90°C. Protein separation was performed using 10% sodium dodecyl sulfate‐polyacrylamide gel electrophoresis, and the proteins were transferred to polyvinylidene difluoride (PVDF) membranes (Millipore, Burlington, MA, USA) using the Bio‐Rad wet transfer system. The membranes were blocked in TBS‐T containing 5% skim milk for 30 min, washed with TBS‐T, and then incubated with primary antibodies (Table S1) overnight at 4°C. Subsequently, the membranes were washed thrice with TBS‐T, and the blots were incubated with anti‐mouse or anti‐rabbit IgG horseradish peroxidase (HRP)‐conjugated secondary antibodies (GeneTex, Irvine, CA, USA) for 1 h at RT. The membranes were washed with TBS‐T and developed using an enhanced chemiluminescence (ECL) solution (Thermo Fisher Scientific).

### 2.8 Human specimens

Human blood plasma samples (*n* = 125) were acquired from healthy individuals and patients with early‐ and late‐stage AD at the Chungbuk National University Hospital Biobank (Cheongju, Korea) and the Korea Biobank Network (Yongin, Korea). The participants signed an informed consent form, and all protocols were approved by the Institutional Review Board of the National Biobank of Korea. All groups comprised individuals aged 54–90 years without distinction of sex. The groups of patients with early‐ and late‐stage AD were defined using mini‐mental state examination scoring (late <16, 16≤ early ≤23, 24≤ healthy). All patients involved in this study signed an informed consent form, and all protocols were approved by the Institutional Review Board of the National Biobank of Korea (approval number 23‐04).

### 2.9 Machine learning

A support vector machine (SVM) was adopted as a classifier to separate datasets and assess the performance of the putative biomarker proteins in AD diagnosis (Yu, 2005). Three classification models for healthy versus early AD, early AD versus late AD, and healthy versus late AD were constructed and validated. This was necessary, as conventional SVM is designed for binary classification.

All features were required to include protein markers that were fully registered in each class (healthy, early, and late AD). The selected features were accumulated through *t*‐test based scoring. Finally, nine proteins were included with common intersections among all classes (Figure S1). Classification accuracy was evaluated using 10 × 10‐fold cross‐validation (Sherman et al., 2022). The classification performance was evaluated using the area under the curve (AUC) and receiver operating characteristic (ROC) curve. The AUC‐ROC curve indicates a possible relationship between sensitivity and specificity for the overall tests (Mandrekar, 2010). All ML processes were performed using MATLAB R2019b (MathWorks, Inc., Natick, MA, USA).

### 2.10 Statistical analysis

Data are expressed as mean ± standard error of the mean. The significance of intergroup differences was determined using a two‐tailed *t*‐test or one‐way analysis of variance (ANOVA), followed by Bonferroni's multiple comparisons test in Prism ver 9.0 (GraphPad Software, Inc., San Diego, CA, USA). Statistical significance was set at a *p* < 0.05.

## 3 RESULTS

### 3.1 Preparation of multi‐proteomes from 5xFAD mice

**FIGURE 1:**
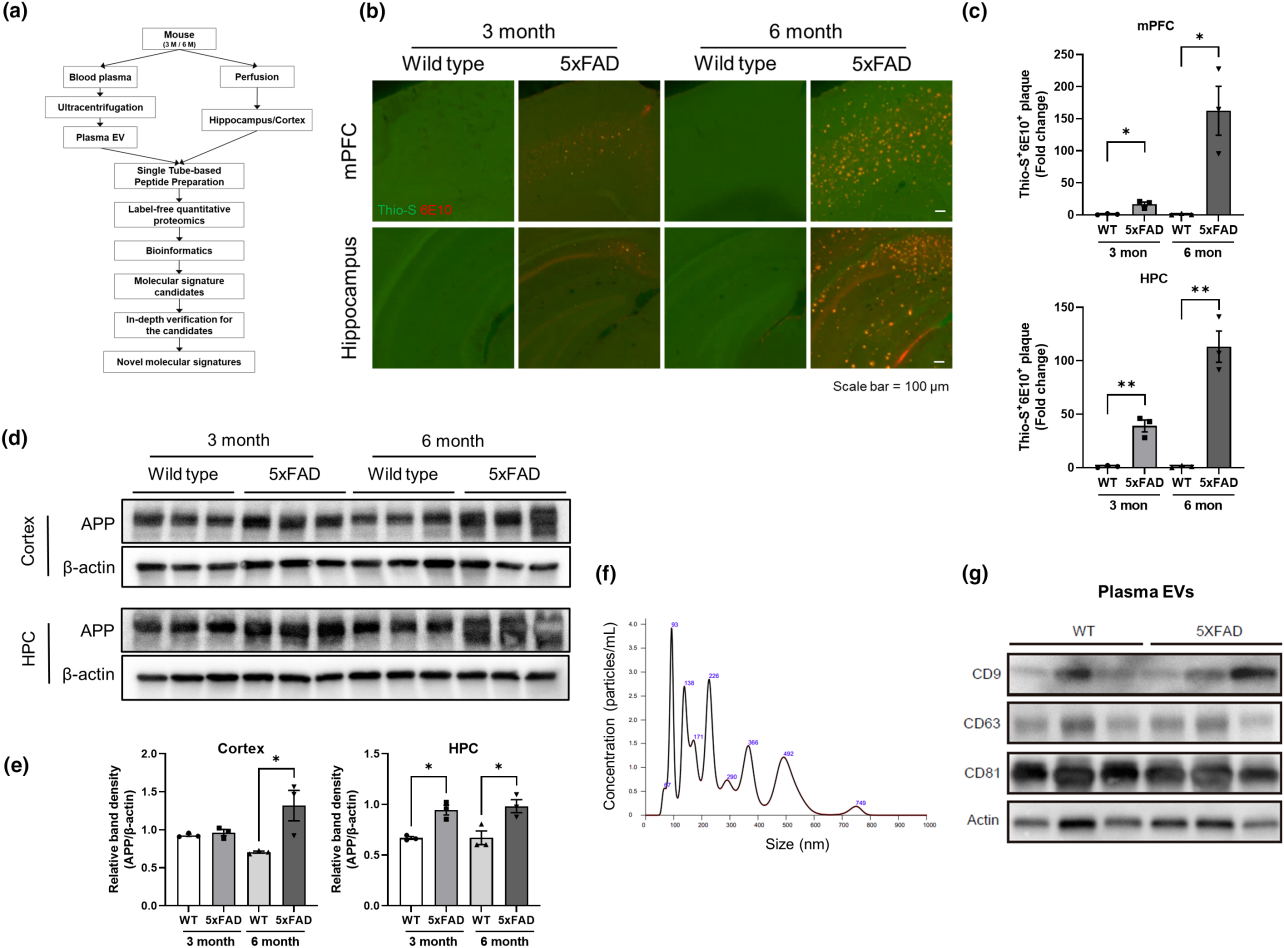
Preparation of multi‐proteomes from wild type (WT) and 5xFAD mice. (a) Study workflow. (b) Stained images using Thioflavin‐S and anti‐Aβ_1‐16_ (clone 6E10) in the medial prefrontal cortex (mPFC) and hippocampus (HPC) of WT and 5xFAD mice. Scale bar = 100 μm. (c) Densitometric graphs of the immunostained images in (b). Data are presented as mean ± standard error (SE) (*n* = 3/group). *p < 0.03, **p < 0.01, and ***p < 0.001 in analysis of variance (ANOVA) with Bonferroni's multiple comparisons. (d) Western blotting of amyloid precursor protein (APP) in brain lysates of WT and 5xFAD mice. (e) Densitometric graphs of western blotting in d. Data are presented as mean ± SE (*n* = 3/group). *p < 0.03 in ANOVA with Bonferroni's multiple comparison test. (f) Nanovesicle‐tracking analysis of plasma extracellular vesicles (EVs). NanoSight LM10 was used to estimate plasma EV size. (g) Western blotting of plasma EV marker proteins. Plasma EVs from WT and 5xFAD mice were electrophoresed and blotted using anti‐cluster of differentiation (CD) 9, anti‐CD63, anti‐CD81, and anti‐actin antibodies.

The workflow presented in Figure 1a was followed to explore novel AD molecular signatures. The Aβ levels were monitored to determine the successful establishment of AD model in the 5xFAD mice. Representative images confirmed the accumulation of Aβ plaques in the medial prefrontal cortex (mPFC) and hippocampus of 3‐month‐old 5xFAD mice, and this was even more pronounced in 6‐month‐old 5xFAD mice (Figure 1b,c; Figures S2 and S3). Only few Aβ plaques were observed in the WT mice. Furthermore, amyloid precursor protein (APP) upregulation was more pronounced in the hippocampus than in the cortex and significantly increased in the cortex and hippocampus of 6‐month‐old 5xFAD mice (Figure 1d,e). Additionally, using an Aβ oligomer‐specific antibody (A11), it was confirmed that the increased Aβ levels observed in the 5xFAD model constituted the aggregated Aβ form (Figure S4).

Subsequently, plasma was isolated from the blood of 5xFAD and WT mice to detect AD‐dependent altered proteins in plasma EVs.

The quality and purity of EVs were estimated using vesicle size analysis; most vesicles were 100 nm in length (Figure 1f). The EV biomarker proteins cluster of differentiation (CD) 9, CD63, and CD81 were highly enriched in plasma EVs (Figure 1g). Collectively, these results indicate that high‐quality plasma EVs from 5xFAD and WT mice were successfully isolated.

### 3.2 Comparative proteomic analyses of multi‐proteomes from WT and 5xFAD mice

**FIGURE 2:**
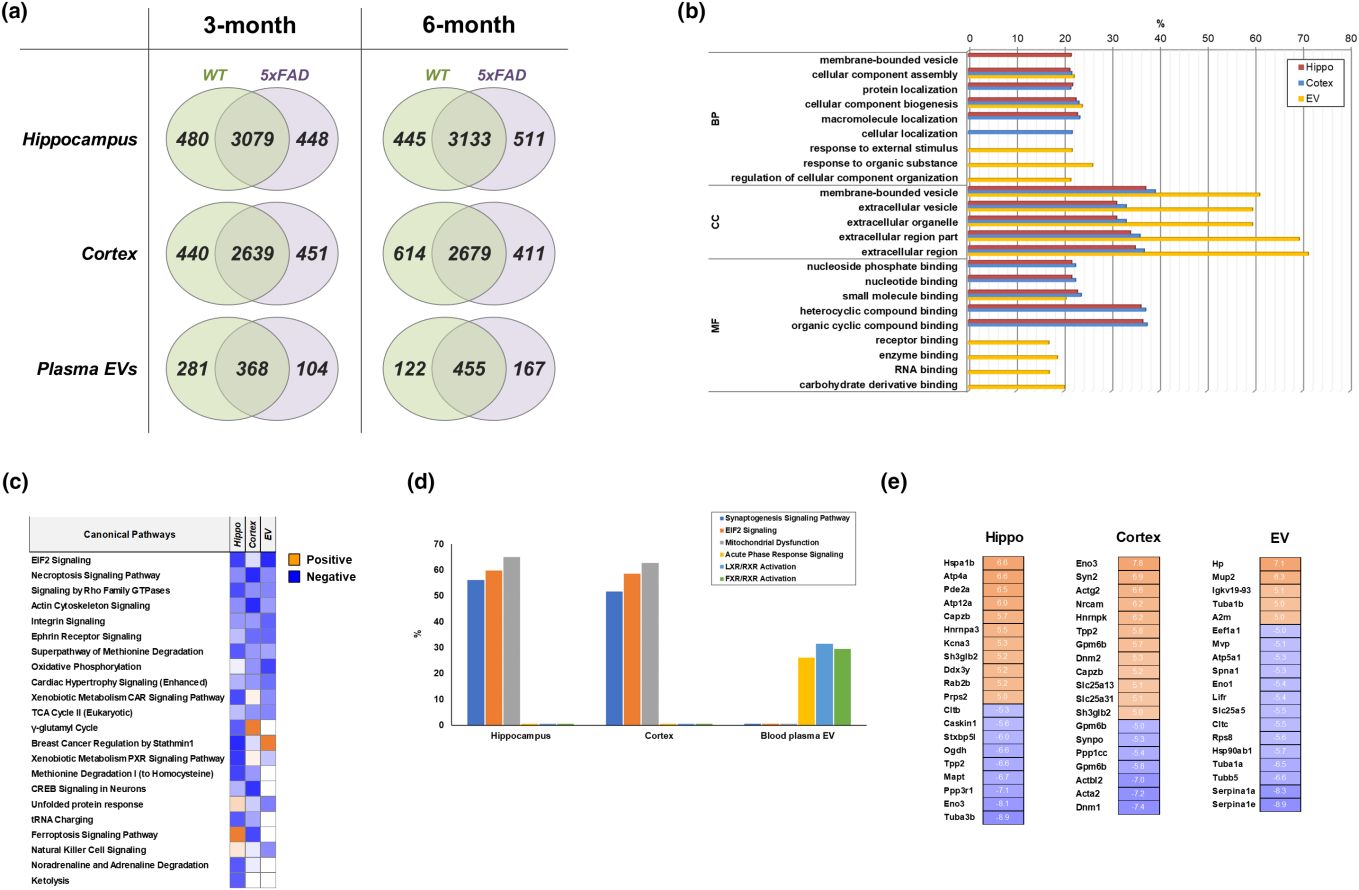
Comparative proteomic analysis between 3‐month‐old WT and 5xFAD mice. (a) Venn diagram of the identified proteins. (b) Enrichment analyses of Gene Ontology (GO)‐based functional annotations in the hippocampus, cortex, and plasma of extracellular vesicles (EVs). (c) Comparative canonical pathway analyses among 3‐month‐old proteomes using Ingenuity Pathway Analysis (IPA). Orange and blue indicate canonical pathways with a positive or negative *Z*‐score, respectively, for pathway activation. Analysis parameters were a *z*‐score cut‐off of 0.5 and a −log (*p*‐value) value of >1.3. (d) Comparative analysis of the top three pathways in the three proteomes. Proteins involved in the top three IPA pathways were identified. (e) Significantly altered proteins in each proteome. Orange and blue indicate a log_2_ fold increase and decrease, respectively.

Proteomic analyses of EVs in 3‐month‐old WT and 5xFAD mice revealed 4007, 3530, and 753 proteins in the hippocampus, cortex, and plasma EVs, respectively (Figure 2a). In 6‐month‐old WT and 5xFAD mice, there were 4089, 3704, and 744 proteins in the hippocampus, cortex, and plasma EVs, respectively (File S1).

Functional annotation enrichment analysis of the hippocampal and cortical proteomes of the 3‐month‐old WT and 5xFAD mice revealed shared GO terms under biological processes (BP), cellular components (CC), and molecular functions (MF) (Figure 2b). The hippocampal and cortical proteomes shared the same cellular component‐related terms and protein localization under GO‐BP, except for membrane‐bound vesicles and cellular localization. Five major terms (EVs, organelles, and their related terms) were common to the hippocampal and cortical proteomes in GO‐CC. The hippocampal and cortical proteomes shared five major terms in GO‐MF: nucleoside phosphate, nucleotides, small molecules, heterocyclic compounds, and organic cyclic compound binding. However, the GO terms and percentage involvement in the proteomes of plasma EV substantially differed from those of the hippocampus and cortex. The plasma EV proteome included unique GO terms, such as response to external stimuli, organic substances, and regulation of cellular component organization in GO‐BP (Figure 2b). The proteomes of plasma EV shared the same GO terms as those of the hippocampus and cortex in GO‐CC. Nevertheless, the involvement percentage of these terms was relatively higher in the proteome of plasma EVs than in those of the hippocampus and cortex. The GO‐MF terms differed between plasma EV and other proteomes.

IPA of the 3‐month‐old proteome dataset revealed that most (13/22) of the canonical pathways with relatively high statistical significance were deactivated in 5xFAD mice (Figure 2c). These included EIF2, necrosis, Rho GTPase, actin, integrin, the ephrin receptor, methionine degradation, oxidative phosphorylation, and the tricarboxylic acid cycle. The hippocampal and cortical proteomes shared all top three IPA canonical pathways, including synaptogenesis, EIF2, and mitochondrial dysfunction. Nevertheless, the plasma EV proteome also included the acute phase response, liver X receptor/retinoid X receptor (LXR/RXR) activation, and farnesoid X receptor (FXR)/RXR activation (Figure 2d). The analysis of the significantly differentially expressed proteins (log_2_fold ≥5) in each proteome revealed that F‐actin capping protein subunit beta (Capzb), endophilin‐B2 (Sh3glb2), tripeptidyl peptidase 2 (Tpp2), and enolase 3 (Eno3) were recapitulated between the hippocampal and cortical proteomes. However, recapitulated proteins were not detected between the plasma EV and other proteomes (Figure 2e). Although only four of the differentially expressed proteins were recapitulated, the hippocampal and cortical proteomes had relatively similar bioinformatics features, as revealed by functional annotation and canonical pathway analyses. Furthermore, most canonical pathways were deactivated in 3‐month‐old 5xFAD mouse brains. The results of the functional annotation and pathway analyses of the plasma EV proteome significantly differed from those of the hippocampal and cortical proteomes.

**FIGURE 3:**
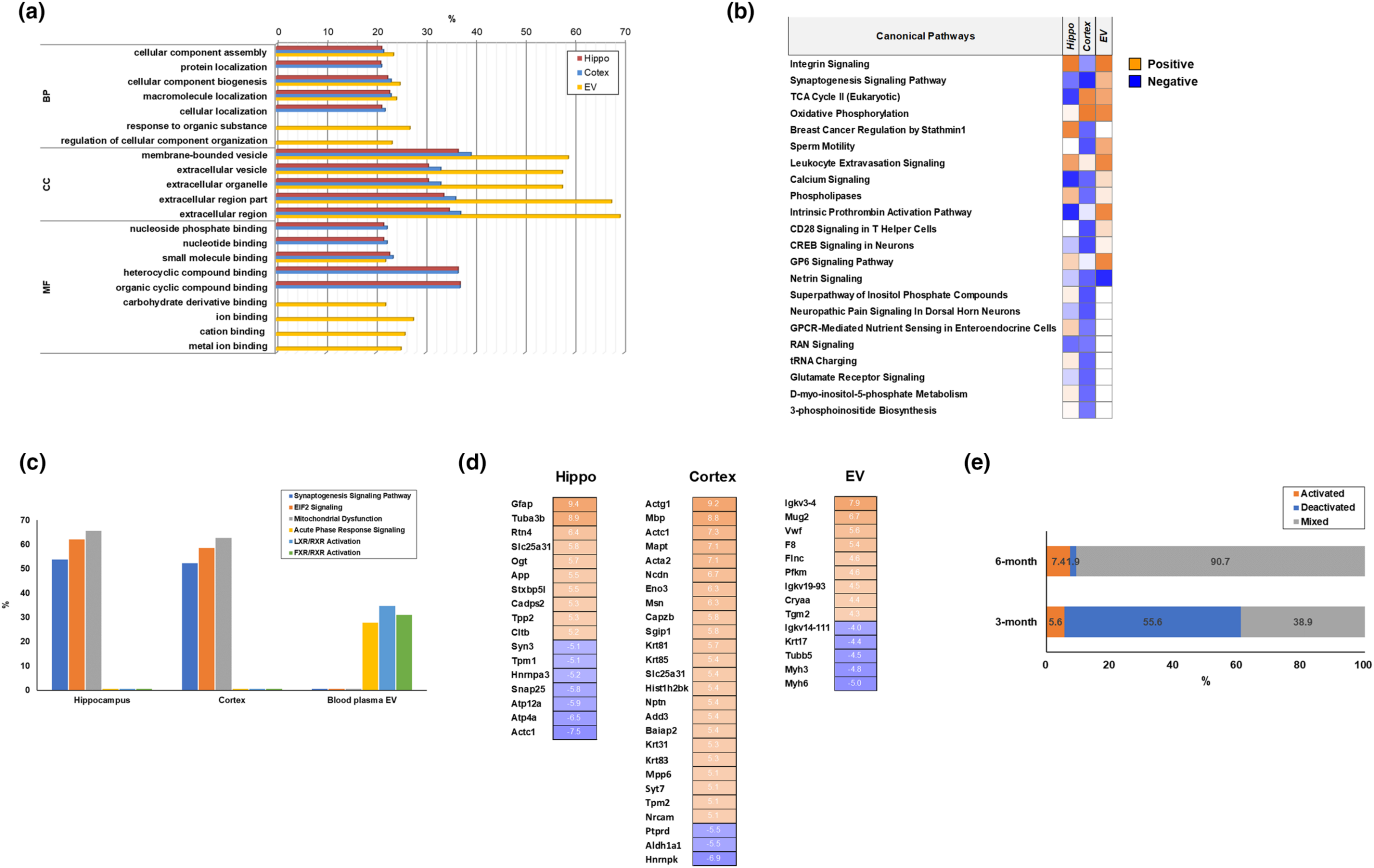
Comparative proteomic analysis between 6‐month‐old WT and 5xFAD mice. (a) Enrichment analysis of GO‐based functional annotation of the hippocampus, cortex, and plasma EVs. (b) Comparative canonical pathway analyses of 6‐month‐old proteomes using IPA. Orange and blue indicate canonical pathways with a positive or negative *Z*‐score, respectively, for pathway activation. Analysis parameters were a z‐score cut‐off of 0.5 and a −log (*p*‐value) value of >1.3. (c) Comparative analysis of the top three pathways in the three proteomes. The proteins involved in the top three IPA pathways were extracted. The top pathways were synaptogenic signaling, EIF2 signaling, mitochondrial dysfunction, acute phase response, LXR/RXR activation, and FXR/RXR activation. (d) Significantly altered proteins in each proteome between 6‐month‐old WT and 5xFAD mice. Orange and blue indicate log_2_ fold increase and decrease, respectively. (e) Comparative analysis of activated and deactivated canonical pathways between 6‐month‐old and 3‐month‐old mice. “Activated” and “Deactivated” indicate activated and deactivated canonical pathways, respectively, in 5xFAD mice analyzed using IPA. “Mixed” indicates a pathway demonstrating mixed directions of activation and deactivation for each proteome.

Functional annotation enrichment analysis of the hippocampal and cortical proteomes of the 6‐month‐old WT and 5xFAD mice revealed several shared GO terms in BP, CC, and MF (Figure 3a). The involvement of GO terms in the plasma EV proteome differed from that in the others, in terms of categories and percentages. The plasma EV proteome included the same new GO terms as the 3‐month‐old plasma EV proteomes (Figure 3a). There were substantial differences between plasma EV and other proteomes under GO‐MF.

The 6‐month‐old proteome had fewer canonical pathways with the same direction of activation/deactivation than the 3‐month‐old proteomes, as indicated using IPA. Only oxidative phosphorylation, leukocyte extravasation, and netrin signaling were in the same direction in all three proteomes (Figure 3b). A comparison of the top three IPA canonical pathways revealed similar results for 3‐ and 6‐month‐old proteomes. The plasma EV proteome substantially differed from the hippocampal and cortical proteomes, which shared all three pathways (Figure 3c). A comparison of the significantly differentially expressed proteins revealed that glial fibrillary acidic protein (Gfap) and solute carrier family 25 member 31 (Slc25a31) were recapitulated between the hippocampal and cortical proteomes. However, no recapitulated proteins were observed between the plasma EV and other proteomes (Figure 3d). Pathway analyses detected significantly different pathway activation/deactivation between the 3‐ and 6‐month‐old mouse profiles. Therefore, the activation profiles of all 54 IPA canonical pathways were analyzed. The 6‐month‐old proteomes had approximately 7.4% pathway activation, 1.9% deactivated pathways, and 90.7% mixed activation pattern (Figure 3e). Conversely, the 3‐month‐old proteomes presented with 55.6% deactivated, 5.6% activated, and 38.9% mixed activation pattern pathways.

Functional annotations of the proteomes of the 6‐month‐old hippocampus and cortex were similar. However, the results of the canonical pathway analyses significantly differed among the three proteomes.

The functional annotation and pathway analyses of the plasma EV proteome significantly differed from those of other proteomes. Therefore, most canonical pathways were highly deactivated in the hippocampus, cortex, and plasma EV proteomes of 3‐month‐old 5xFAD mice but not in those of 6‐month‐old 5xFAD mice.

### 3.3 Comparative analysis of the multi‐proteomes of 3‐ and 6‐month‐old 5xFAD mice

**FIGURE 4:**
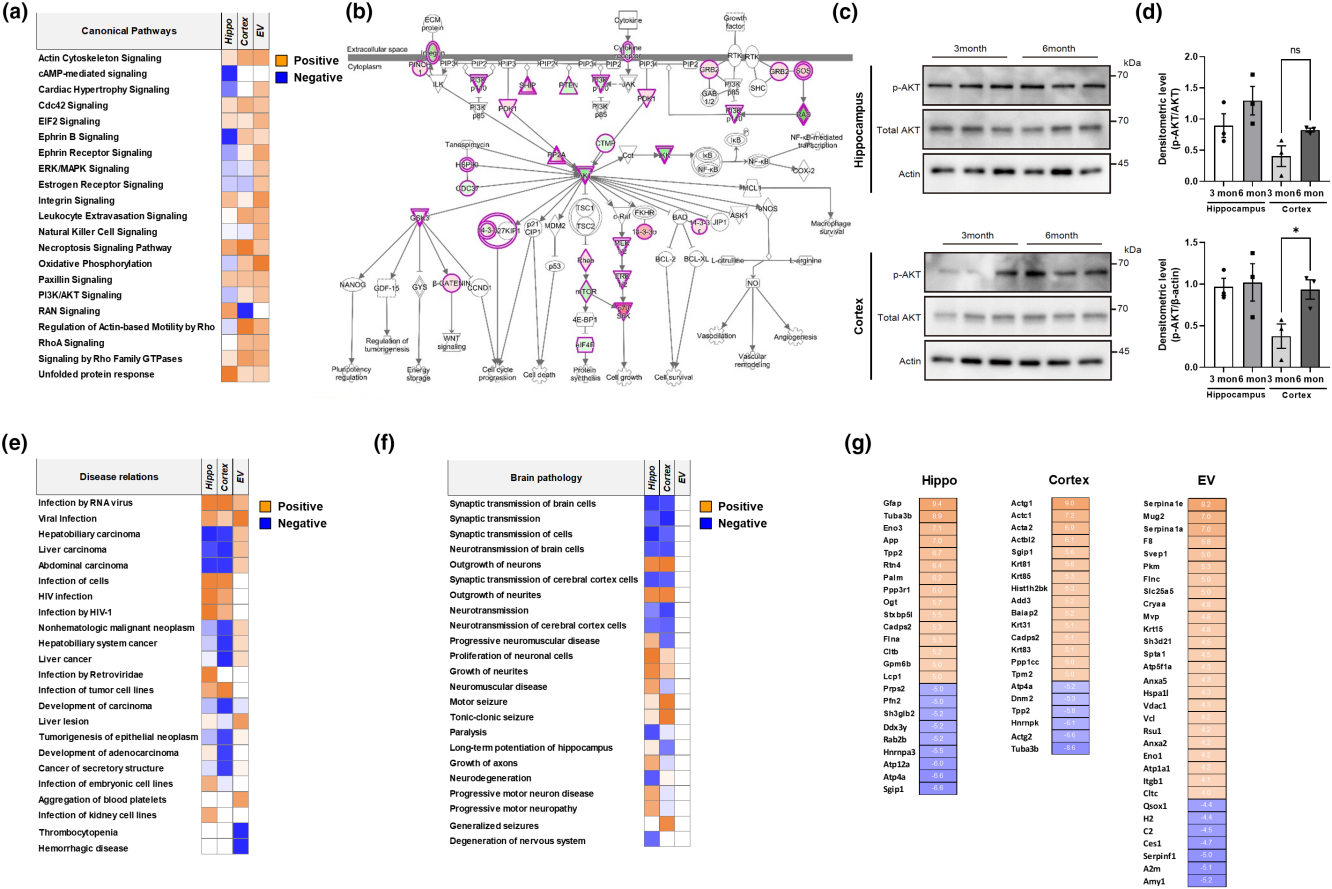
Comparative bioinformatics analysis between 3‐ and 6‐month‐old 5xFAD mice. (a) Comparative canonical pathway analysis of the three proteomes using IPA. Orange and blue indicate canonical pathways with a positive or negative *Z*‐score, respectively, for pathway activation. Analysis parameters were a *z*‐score cut‐off value of 0.5 and a −log (*p*‐value) value of >1.3. (b) IPA of phosphoinositide 3‐kinase/protein kinase B (PI3K/Akt) signaling in the hippocampal proteome. Red and green indicate upregulation and downregulation, respectively, in the 6‐month‐old proteome, compared with the 3‐month‐old proteome. Yellow arrows indicate contrasting expression patterns of signaling proteins in the hippocampal and cortical proteomes. (c) Western blotting of Akt (S473) phosphorylation in the hippocampi and cortices of 3‐ and 6‐month‐old 5xFAD mouse brains. The tissue lysates were electrophoresed and blotted with each antibody. Actin was used as a loading control. (d) Densitometric graphs of western blotting in “c”. Data are presented as mean ± SE (*n* = 3/group). *p < 0.03 in ANOVA with Bonferroni's multiple comparison test. (e) Comparative disease and biofunction analyses of the three proteomes using IPA. Orange and blue indicate canonical pathways with a positive or negative *Z*‐score, respectively, for pathway activation. (f) Enrichment analysis of brain pathology‐related terms among the three proteomes using IPA. Orange and blue indicate canonical pathways with a positive or negative *Z*‐score, respectively, for pathway activation. (g) Significantly altered proteins in the proteomes of 3‐ and 6‐month‐old 5xFAD mice. Orange and blue indicate a log_2_ fold increase and decrease, respectively.

Functional annotation analysis revealed that the hippocampal and cortical proteomes shared BP, CC, and MF GO terms (Figure S5). The 3‐ and 6‐month‐old proteomes shared cellular component‐related terms and protein and macromolecule localization under GO‐BP. The results for the 3‐month‐old proteomes were recapitulated with those of the analyses of the 6‐month‐old hippocampal and cortical proteomes under GO‐CC and GO‐MF. The categories and percentages of involvement of the GO terms were distinct in the plasma EV proteomes. The 3‐ and 6‐month‐old plasma EV proteomes contained unique GO terms (Figure S5). Substantial differences were observed between the plasma EV and other proteomes under GO‐MF. Canonical pathway analysis revealed that both 3‐ and 6‐month‐old hippocampal proteomes presented relatively more downregulated pathways than the other proteomes (Figure 4a). The deactivated pathways in the hippocampal proteomes included the cAMP‐mediated, ephrin‐related, ERK, estrogen receptor, and PI3K/Akt signaling pathways. Several reports suggest that these results are actually in line with AD pathology. For instance, it has been reported that Aβ leads to the inactivation of protein kinase A, a downstream molecule of cAMP (Vitolo et al., 2002), and that overexpression of β‐site APP‐cleaving enzyme 1, increased levels of which contribute to the pathogenesis of sporadic AD, reduces cAMP levels (Chen et al., 2012). In the context of ephrin‐related pathways, it has been reported that Aβ reduces Eph receptor levels and that these Eph receptors play a role in processes related to neuronal dysfunction in AD (Dines & Lamprecht, 2016). Additionally, the depletion of EphB2 receptor caused by Aβ oligomers could impact its downstream signaling, leading to impairments in synaptic plasticity in the hippocampus (Cissé & Checler, 2015). The relationship between estrogen and several functions of the central nervous system has also been revealed (Mosconi et al., 2018; Scheyer et al., 2018). Furthermore, several previous studies have suggested that the loss of estrogen and reduction of levels of estrogen receptors in the brain increases the incidence of AD (Rettberg et al., 2014). The cortical proteome exhibited relatively fewer deactivated pathways than the hippocampal proteome. The proteome analysis revealed substantially different activation patterns across ephrin‐related signaling, oxidative phosphorylation, PI3K/Akt signaling, and actin‐based mobility, all induced by Rho. Only the Ras‐related nuclear protein signaling pathway was deactivated in the cortical proteome. All canonical pathways were activated in an age‐dependent manner in the plasma EV proteomes. These analyses revealed a major AD lesion site. This indicated that disease severity is associated with the hippocampus rather than with the cortex.

We selected PI3K/Akt signaling for subsequent canonical pathway analysis based on the observation that its regulation pattern in the hippocampal proteome differed from that in the cortical proteome. The expression levels of 21/59 signaling proteins in the PI3K/Akt pathway (including Akt and PI3K) significantly differed between the hippocampal and cortical proteomes (Figure 4b). Western blotting indicated that phospho‐Akt levels in the 3‐month‐old 5xFAD cortex were lower than those in the 6‐month‐old 5xFAD cortex tissues (Figure 4c,d), as corroborated using canonical pathway analysis.

Hepatobiliary, liver, and abdominal carcinomas and several neoplasms negatively correlated with progression of AD pathology in 5xFAD mice (Figure 4e). The associations between plasma EV proteomes and other diseases were distinct from those of other proteomes. Most diseases positively correlated with plasma EV proteomes in an age‐dependent manner. Synaptic transmission‐related terms in the hippocampal and cortical proteomes negatively correlated with progression of AD pathology in 5xFAD mice (Figure 4f). Published literature suggests that this is consistent with AD pathology. For instance, through previous studies of the post‐mortem brain of AD patients and mouse models, it has been discovered that Aβ oligomers accumulate within synapses, resulting in an extensive loss of excitatory synapses (Tzioras et al., 2023). Furthermore, microarray analysis revealed significantly decreased expression of synaptic genes in the hippocampal regions of AD patients compared with that in age‐matched controls (Berchtold et al., 2013). In contrast, neuronal outgrowth, proliferation, and seizure‐related terms positively correlated with progression of AD pathology in 5xFAD mice. Nevertheless, the correlation between the plasma EV proteome and brain pathology was weak (Figure 4f). Tubulin alpha 3b (Tuba3b), Tpp2, calcium‐dependent secretion activator 2 (Cadps2), ATPase H^+^/K^+^ transporting subunit alpha (Atp4a), and SH3GL interacting endocytic adaptor 1 (Sgip1) were recapitulated in the hippocampal and cortical proteomes (Figure 4g). However, no recapitulated proteins were observed between the plasma EV and other proteomes.

A comparison of the age‐dependent 5xFAD proteome revealed that the hippocampal proteome contained highly deactivated pathways, suggesting that the hippocampus is the major lesion site that reflects AD severity. Various cancers negatively correlated with progression of AD pathology in 5xFAD mice. Synaptic transmission‐related terms negatively correlated with progression of AD pathology in the hippocampal and cortical proteomes of 5xFAD mice. These findings facilitated the identification of the molecular signatures that could serve as a theoretical basis to elucidate AD pathology. Novel candidate AD biomarkers were selected among the upregulated proteins detected in 5xFAD mice (Table 1).

**TABLE 1:** Significantly changed proteins in 5xFAD mice.

#	Description	Gene ID	3‐month‐old EV		6‐month‐old EV		3‐month‐old HPC		6‐month‐old HPC		3‐month‐old Ctx		6‐month‐old Ctx		Clinical trial
			Log_2_ fold	*p*‐value	Log_2_ fold	*p*‐value	Log_2_ fold	*p*‐value	Log_2_ fold	*p*‐value	Log_2_ fold	*p*‐value	Log_2_ fold	*p*‐value	
1	Integrin alpha‐IIb	*Itga2b*	−1.3	0.0001	0.6	0.0253	−0.6	0.6532	−2.6	0.1829	−1.0	0.6799	0.2	0.9273	X
2	Talin‐1	*Tln1*	−1.3	0.0000	0.6	0.0108	0.7	0.1953	1.0	0.0296	0.2	0.6959	0.2	0.7239	X
3	Filamin‐A	*Flna*	−2.0	0.0000	0.5	0.0174	ND	ND	0.7	0.2041	−3.0	0.0191	3.4	0.0062	X
4	Integrin alpha‐6	*Itga6*	−2.7	0.0005	1.1	0.0304	ND	ND	ND	ND	ND	ND	ND	ND	X
5	Protein‐glutamine gamma‐glutamyltransferase 2	*Tgm2*	−3.8	0.0000	4.3	0.0042	−0.6	0.6532	0.5	0.5584	0.0	0.9960	0.2	0.8745	X
6	Major urinary protein 2	*Mup2*	6.3	0.0000	4.0	0.0109	ND	ND	ND	ND	ND	ND	ND	ND	X
7	Alpha‐1‐acid glycoprotein 2	*Orm2*	4.3	0.0029	2.8	0.0335	ND	ND	ND	ND	ND	ND	ND	ND	X
8	Murinoglobulin‐2	*Mug2*	ND	ND	6.7	0.0000	ND	ND	ND	ND	ND	ND	ND	ND	X
9	Filamin C	*Flnc*	ND	ND	4.6	0.0010	−1.0	0.6823	ND	ND	ND	ND	ND	ND	X
10	ATP‐dependent 6‐phosphofructokinase, muscle type	*Pfkm*	ND	ND	4.6	0.0010	0.0	0.8533	0.1	0.6199	0.0	0.7990	0.0	0.9104	X
11	Isoform 2 of alpha‐crystallin A chain	*Cryaa*	ND	ND	4.4	0.0026	ND	ND	ND	ND	ND	ND	ND	ND	X
12	Heat shock 70‐kDa protein 1‐like	*Hspa1l*	ND	ND	4.0	0.0109	0.0	0.9405	0.2	0.3304	0.0	0.9587	−0.1	0.7915	X
13	Voltage‐dependent anion‐selective channel protein 1	*Vdac1*	ND	ND	4.0	0.0109	0.1	0.4452	−0.1	0.5607	0.0	0.8182	−0.2	0.1824	X
14	Creatine kinase M‐type	*Ckm*	ND	ND	3.8	0.0177	−0.1	0.8240	0.4	0.5060	0.0	0.9887	0.2	0.8232	X
15	Alpha‐2‐macroglobulin‐P	*A2m*	5.0	0.0001	ND	ND	ND	ND	ND	ND	ND	ND	ND	ND	X
16	Isoform 3 of sulfhydryl oxidase 1	*Qsox1*	4.3	0.0029	ND	ND	ND	ND	ND	ND	ND	ND	ND	ND	X
17	Haptoglobin	*Hp*	7.1	0.0000	ND	ND	1.0	0.6839	−2.6	0.0596	1.0	0.6863	−3.1	0.0765	O
18	H‐2 class I histocompatibility antigen, L‐D alpha chain	*H2‐L*	4.3	0.0029	ND	ND	ND	ND	ND	ND	ND	ND	ND	ND	X
19	Phospholipid transfer protein	*Pltp*	3.5	0.0036	ND	ND	ND	ND	−1.0	0.6852	ND	ND	ND	ND	X
20	Lysosomal alpha‐mannosidase	*Man2b1*	3.3	0.0057	ND	ND	ND	ND	ND	ND	ND	ND	ND	ND	X
21	Platelet factor 4	*Pf4*	2.4	0.0000	−3.2	0.0000	ND	ND	ND	ND	ND	ND	ND	ND	X

Abbreviations: Ctx, cortex; EV, extracellular vesicle; HPC, hippocampus; ND, not determined.

### 3.4 Validation of biomarker candidate proteins using plasma EVs from stage‐divided patients with AD

Plasma EVs, cortices, and hippocampi from 3‐month‐old 5xFAD mouse tissues were subjected to western blotting to identify potential AD biomarkers (Figure S6). CD63 was confirmed as an EV marker, and platelet integrin receptor αIIb‐β3 (Itga2b), voltage‐dependent anion channel (Vdac), mannosidase alpha class 2B member 1 (Man2b1), quiescin sulfhydryl oxidase 1 (Qsox1), alpha‐2‐macroglobulin (A2m), transglutaminase 2 (Tgm2), and phospholipid transfer protein (Pltp) were upregulated in the plasma EVs of 3‐month‐old 5xFAD mice (Figure S6a). Only Man2b1 showed a significant change in expression in the cortex and hippocampus (Figure S6b,c).

**FIGURE 5:**
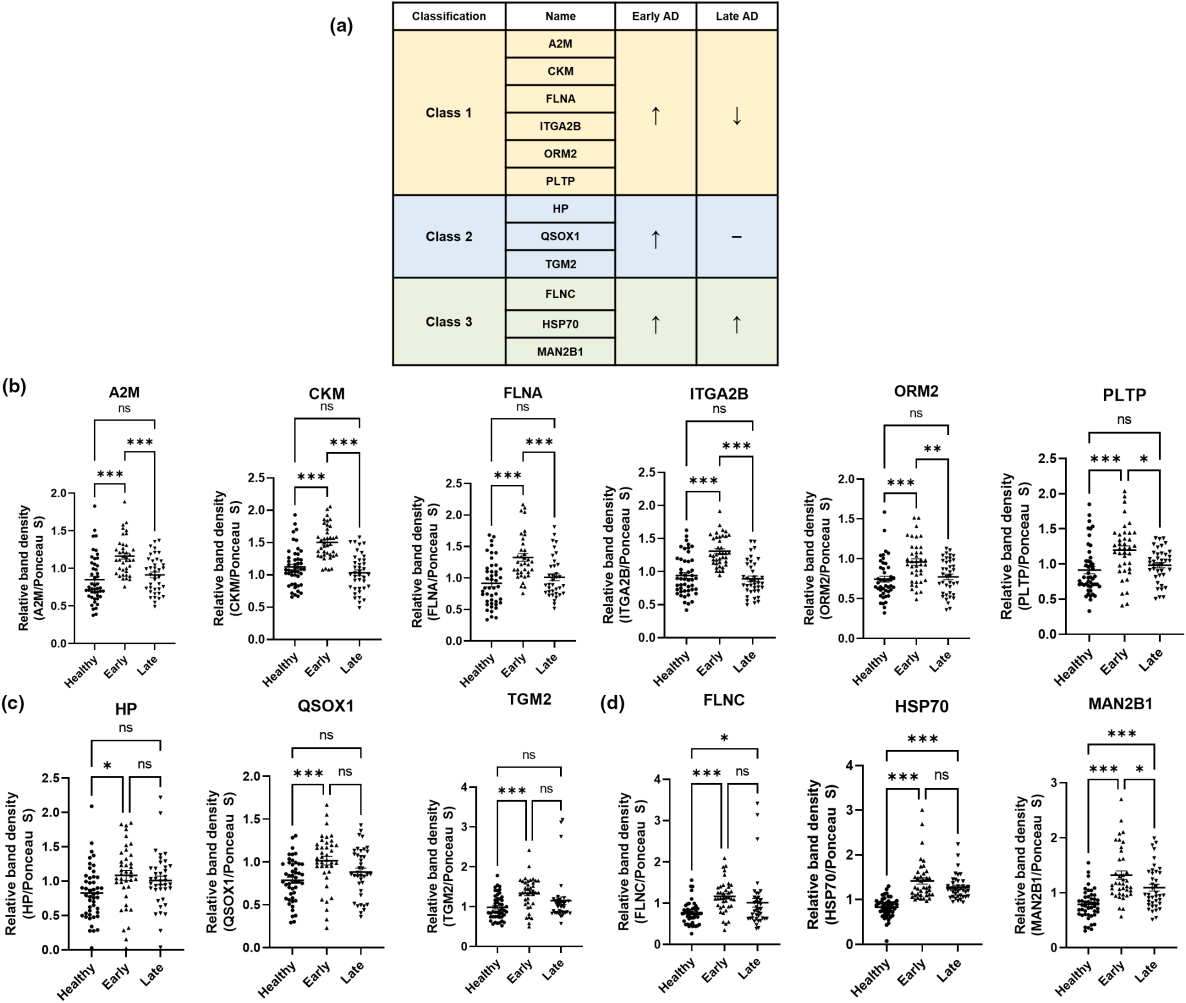
Validation of biomarker candidate proteins using plasma EVs from stage‐divided patients with AD. Western blotting of selected biomarker candidates in plasma EVs from healthy individuals and patients with early‐ and late‐stage AD. Plasma EVs were electrophoresed and blotted with antibodies. “Early” and “late” stages of AD were diagnosed using mini‐mental state examination scoring. (a) Classification according to the results of western blotting. (b) Scatterplot of class 1 proteins, including A2M, CKM, FLNA, ITGA2B, ORM2, and PLTP. (c) Scatterplot of class 2 proteins, including HP, QSOX1, and TGM2. (d) Scatter plot of class 3 proteins, including FLNC, HSP70, and MAN2B1. All results were densitometrically analyzed using ImageJ Ver 1.53 after normalization to the density of bands stained with Ponceau S. ***p < 0.001, **p < 0.01, and *p < 0.03 in one‐way ANOVA with Bonferroni's multiple comparisons (*n* = 39–47/group). ns, not significant.

We attempted to validate the potential utility of the 14 AD biomarker candidates in patients with AD. To this end, 39–47 plasma specimens were collected from each group of healthy individuals and patients with early‐ and late‐stage AD, and each specimen was classified according to mini‐mental state examination scores (late <16, 16≤ early ≤23, 24≤ healthy) (File S2). Human plasma EVs were isolated, and the presence of EV markers (such as ALIX, CD9, and CD63) was confirmed (Figures S7 and S8) and validated using western blotting. As expected, Tau protein levels tended to increase in both patients with early‐ and late‐stage AD (Figure S9). Twelve biomarker candidates that were upregulated in the plasma EVs of patients with early‐stage AD were identified and sorted into three classes according to their expression patterns in late‐stage AD (Figure 5a). The levels of class 1 proteins (A2M, CKM, FLNA, ITGA2B, orosomucoid 2 [ORM2], and PLTP) significantly increased in patients with early‐stage AD. In contrast, they did not change in patients with late‐stage AD relative to those in healthy individuals (Figure 5b and Figure S10a–f). The levels of class 2 proteins (HP, QSOX1, and TGM2) significantly changed in patients with early‐stage AD, compared with those in healthy individuals (Figure 5c; Figure S10g–i). The levels of class 3 proteins (FLNC, HSP70, and MAN2B1) significantly increased in patients with early‐ and late‐stage AD compared with those in healthy individuals (Figure 5d; Figure S10j–l). PF4 and TLN1 levels exhibited high individual differences and did not differ between the groups (Figures S11 and S12). These results indicate that the levels of the selected candidates (except PF4 and TLN1) are distinguishable from those of healthy individuals. Furthermore, class 1 proteins were considered unique diagnostic biomarkers of early‐stage AD.

### 3.5 Performance testing using ML

**FIGURE 6:**
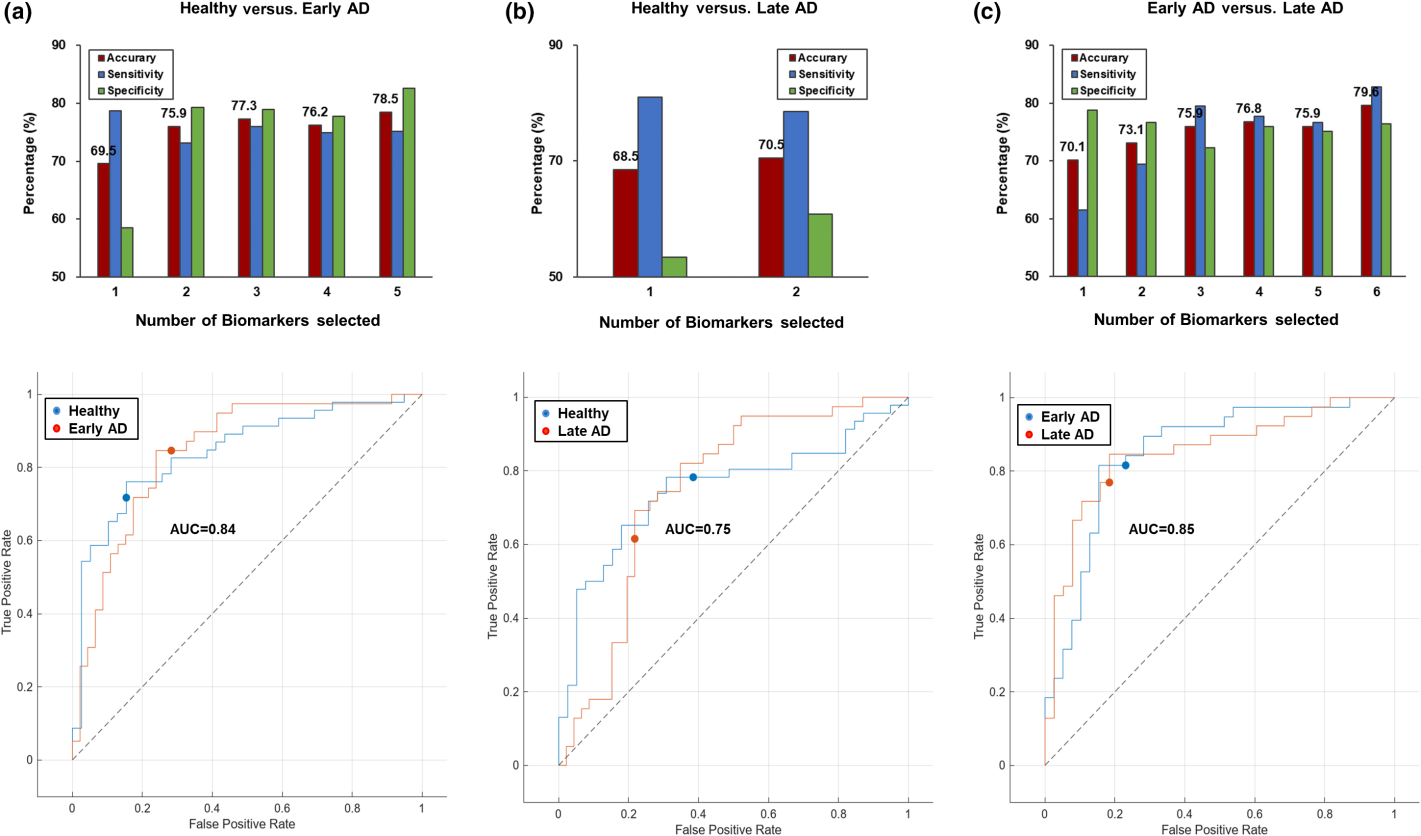
Performance test of the proposed ML model based on the AUC‐ROC curve. Accumulated accuracy, sensitivity (SEN), specificity (SPE), and AUC‐ROC curves for (a) healthy versus early‐stage AD, (b) healthy versus late‐stage AD, and (c) early‐stage AD versus late‐stage AD. The bottom figures show the AUC‐ROC curves of the selected features.

An ML model was employed to validate the performance of the selected biomarkers in distinguishing between patient groups. The performance levels of the common intersection proteins (including ITGA2B, CKM, FLNC, MAN2B1, TGM2, A2M, FLNA, ORM2, and PLTP) were assessed. The best classification performance in the SVM classifiers for healthy versus early‐stage AD was 78.5% when five protein features (ITGA2B, FLNC, CKM, TGM2, and MAN2B1) were layered (Figure 6a). The highest accuracy was 79.6% with the six‐layered features of comparison between early‐ and late‐stage AD (CKM, ITGA2B, A2M, ORM2, PLTP, and FLNA; Figure 6c). Our proposed model showed a reasonably accurate classification rate of 70.5% for healthy versus late‐stage AD (MAN2B1 and FLNC) (Figure 6b). The classification performance of healthy versus early‐stage AD was validated with an AUC of 0.84 (Figure 6a). The areas under the curve in healthy versus late and early versus late were 0.75 and 0.85, respectively (Figure 6b,c), with values >0.8 indicating excellent performance.

## 4 DISCUSSION

Our findings revealed possible new molecular signatures of progression of AD pathology and identified differentially regulated pathways between the hippocampus and cortex, such as PI3K/Akt signaling. Furthermore, most canonical pathways in the hippocampal and cortical proteomes were deactivated in 3‐month‐old 5xFAD mice (Figure 2c) but not in 6‐month‐old 5xFAD mice. The proteomes of 3‐month‐old 5xFAD mice contained 55.6% deactivated canonical pathways (Figure 3e), whereas only 1.9% of the canonical pathways were deactivated in the proteomes of 6‐month‐old 5xFAD mice. Our findings (Figure 1b,c), which are in line with those of previous studies, suggested that the 5xFAD mice presented with an extensive accumulation of Aβ in their cortical and hippocampal lesions; this pathological process may alter brain homeostasis (Oakley et al., 2006). The attenuation of signaling pathways actively occurred in 3‐month‐old 5xFAD mice but not in 6‐month‐old 5xFAD mice.

An early diagnosis of AD is necessary and challenging. Earlier medical interventions showed better treatment effects and delays in dementia (Rasmussen & Langerman, 2019). Because of the importance of understanding AD pathogenesis alongside an early AD diagnosis, we compared our proteomic results with those of a previous study that conducted proteomic analysis in neonatal 5xFAD mice (Mazi et al., 2018). Compared with our study, the previous study showed several common results in the observed altered signaling pathways, including signaling by Rho family GTPases in the 3‐month‐old group (Figure 2c), integrin signaling and netrin signaling in the 6‐month‐old group (Figure 3b), and ephrin‐related signaling, integrin signaling, and signaling by Rho family GTPases in the comparison between 3‐month‐old and 6‐month‐old datasets (Figure 4a). Additionally, it could be considered that molecular and cellular functions related to cellular assembly and organization, cellular function and maintenance, and cell morphology were altered in both the cortical and hippocampal regions of 5xFAD mice. The top altered canonical signaling pathways identified using the IPA included synaptogenesis, EIF2, and mitochondrial dysfunction in 3‐ and 6‐month‐old 5xFAD mice; these were identified only in our study. These differences were observed in comparison with the progression of AD pathology. Furthermore, we focused on proteomic datasets related to brain pathology in progression of AD pathology to select biomarker targets. Therefore, the main reason for the differences between the two studies is that we conducted a study to discover the proteomic changes caused by the progression of AD pathology using 3‐ and 6‐month‐old mice, whereas the previous study used only neonatal mouse brains. In addition, other factors including experimental methods and informatics criteria might have contributed to these differences. As there is significant interest in the pathogenesis and diagnosis of AD, conducting further proteomic studies related to the molecular mechanism of AD is essential. The present study used a reliable proteomic approach to analyze sub‐proteomes to identify novel biomarkers that can diagnose early‐stage AD. Western blotting using human plasma EVs showed 12 upregulated proteins in patients with AD compared with healthy individuals (Figure 5a). Notably, A2M, CKM, FLNA, ITGA2B, ORM2, and PLTP levels significantly increased in patients with early‐stage AD but remained unchanged in patients with late‐stage AD (Figure 5b), and these proteins were classified as class 1. A2M is a plasma protein structurally and functionally resembling alpha‐macroglobulins and is synthesized in the brain (Tian et al., 2021). A common variant of A2M increases the risk of AD (Tian et al., 2021). A2M is localized in diffuse amyloid plaques, binds to soluble Aβ, and mediates its degradation in the brains of patients with AD. However, excessive A2M has neurotoxic effects, and its abundance in the CSF and blood has been previously confirmed; clinical evidence demonstrates that A2M plays a critical role in AD etiopathology (Kovacs, 2000; Varma et al., 2017). We observed substantially increased A2M levels in the plasma EVs of patients with early‐stage AD.

Creatine is regulated by creatine kinase (CK) and plays a vital role in maintaining energy homeostasis in the brain (Hemmer & Wallimann, 1993). Abnormal CK function is detected in the AD brain, and elevated creatine levels are detected in APP‐transgenic mice and postmortem AD human brains (Gallant et al., 2006). Our data indicated that CKM levels were significantly elevated in plasma EVs from patients with early‐stage AD. Filamin is a homodimeric actin‐binding protein with three isoforms. Filamins A and B are co‐expressed within neurons, and mutations in the X‐linked gene *FLNA* lead to periventricular heterotopia, which is characterized by failed neuronal migration into the cerebral cortex during development (Sheen et al., 2002). Furthermore, altered FLNA induced a pathogenic signaling pathway of Aβ through the α7 nicotinic acetylcholine receptor to activate kinases that hyperphosphorylate tau (Burns & Wang, 2017). We observed upregulated FLNA and FLNC levels in the plasma EVs of patients with AD. Integrins are transmembrane glycoprotein signaling receptors that bidirectionally transmit information and traverse plasma membranes (Youmans et al., 2012). ITGA2B binds to the N‐terminal residues of Aβ and induces outside‐in signaling and Aβ fibril formation (Huang et al., 2019). Aβ and the integrin αIIb–β3 complex generate a feedforward loop promoting Aβ aggregation (Huang et al., 2019). To the best of our knowledge, the present study is the first to identify ITGA2B as a biomarker for early‐stage AD.

Orosomucoid 2 (ORM2) is mostly synthesized by the liver, secreted into the plasma, and belongs to the immunocalin family with immunomodulatory functions (Jo et al., 2017). Our results are consistent with those of previous studies reporting that the levels of ORM are elevated in the sera of patients with central fatigue syndrome and depression, indicating its potential involvement in regulating cognitive function (Adeoye et al., 2003; Sun et al., 2016). PLTP is a complex glycosylated protein that plays a crucial role in lipid metabolism by transporting phospholipids, cholesterol, diacylglycerides, apolipoproteins, and tocopherols (Wang et al., 2021). It is highly expressed in the brain and is affected by various brain functions. PLTP deficiency impairs Aβ clearance through autophagic dysfunction and accelerates memory dysfunction in APP/PS1 mice (Tong et al., 2015). In contrast, the PLTP levels in the plasma EVs of patients with early‐stage AD were significantly higher than those of healthy individuals.

The levels of class 2 proteins (HP, QSOX1, and TGM2) significantly increased in patients with early‐stage AD (Figure 5c). HP is abundant in plasma, and it plays a vital role in the clearance of hemoglobin through strong noncovalent bonds with free hemoglobin and decreases oxidative stress by reducing free radicals (Alayash et al., 2013). Furthermore, HP impairs cholesterol homeostasis by binding to apolipoprotein E (APOE) and forms a complex with Aβ in the brains of patients with AD (Spagnuolo et al., 2014). QSOX1 binds to the endoplasmic reticulum (ER) membrane, catalyzes the formation of disulfide bonds in unfolded proteins (Poillet et al., 2014), and inhibits autophagic flux (Poillet et al., 2014). It may also be involved in neuropathology. Moreover, a proteomic analysis showed that QSOX1 levels increased in the urinary exosomes of 5xFAD mice (Song et al., 2020). TGM2 is a multifunctional enzyme that catalyzes protein crosslinking through lysine isopeptide bonds. It modulates the interactions of the inositol 1,4,5‐triphosphate (IP3) receptor and VDAC1 (D'Eletto et al., 2018). Increased calcium transport is mediated by VDAC1–IP3 receptor bridge formation in primary hippocampal neurons involved in AD (Hedskog et al., 2013). Furthermore, TGM2 inhibition reduces HG‐induced amyloidogenesis in SH–SY5Y neuroblastoma cells (Lee et al., 2021). Therefore, elevated TGM2 levels may serve as a novel biomarker for AD diagnosis.

Class 3 protein (FLNC, HSP70, and MAN2B1) levels were significantly upregulated in patients with early‐ and late‐stage AD (Figure 5d). HSP70 chaperones have a wide range of cellular housekeeping activities, including the folding of newly synthesized proteins and the translocation of peptides into the mitochondria and ER. Moreover, HSP70 responds to various cellular stimuli, such as heat, pressure, ischemia, and hypoxia (Jäättelä, 1999; Rosenzweig et al., 2019). Neuronal damage caused by the misfolding of Aβ and Tau is considered the leading cause of AD, and the importance of chaperones in AD (including HSP70) has been demonstrated (Campanella et al., 2018). MAN2B1 is responsible for N‐linked glycoprotein degradation and is highly expressed in glioma malignancy (Lin et al., 2022). The level of MAN2B1 increased, along with those of immune‐associated proteins, in the CSF proteome of APP/PS1 AD mice and A30P‐αS Parkinson's disease model mice (Eninger et al., 2022). To the best of our knowledge, this is the first study to report elevated MAN2B1 expression levels in the plasma EVs of patients with AD.

Healthy and early‐stage AD were classified with an accuracy of 78.5% and an AUC of 0.84 when the ITGA2B, FLNC, CKM, TGM2, and MAN2B1 proteins were combined using the ML model (Figure 6). The highest level of accuracy achieved was 79.6% (AUC = 0.85) when distinguishing between early‐ and late‐stage AD. Several ML studies used amyloid beta and tau biomarkers obtained from functional brain imaging (Chang et al., 2021). To date, no study has explored the use of an ML model to predict early‐stage AD. This is the first study to perform comparisons between healthy, early‐stage AD, and late‐stage AD using plasma protein biomarkers. The development of an ML model to predict AD diagnosis by identifying protein biomarkers is underway (Mann et al., 2021). Our proposed ML model showed a good performance of up to 79.6% for distinguishing between early‐ and late‐stage AD (Figure 6c), compared with the 70.5% (healthy vs. late) (Figure 6b). This model could aid the discovery of protein biomarkers in patients with early‐ and late‐stage AD.

This study proposed novel biomarkers for the early diagnosis of AD found in the plasma EVs of the 5xFAD mouse model. However, some limitations should be acknowledged. It is necessary to investigate whether changes in plasma EVs are observed in other neurodegenerative diseases and whether such changes are specifically distinguishable from those observed in AD through in‐depth studies. Furthermore, it is worthwhile to compare and analyze the blood‐based biomarkers that are well‐studied in the field of cancer. Nevertheless, combinatorial biomarkers including MAN2B1 are suggested for the first time and demonstrate the potential for practical applications in the clinical stage.

## 5 CONCLUSIONS

In this study, multi‐proteomic analyses revealed molecular signatures that can aid the elucidation of AD pathology. Moreover, our study identified candidate blood plasma EV biomarkers for the diagnosis of early‐stage AD, which included A2M, CKM, FLNA, ITGA2B, ORM2, PLTP, HP, QSOX1, TGM2, FLNC, HSP70, and MAN2B1. According to the ML‐based performance test, the top five biomarkers, namely, CKM, FLNC, ITGA2B, MAN2B1, and TGM2, demonstrated 78.5% accuracy in distinguishing between healthy individuals and those with early‐stage AD.

## AUTHOR CONTRIBUTIONS

J.H.Y. designed the study. S. L., K. I. J., H. L., Y. S. J., D. K., G. P., S. B., and Y. W. K. performed the experiments. S.L., H.L., D.K., Y.S.J., G.P., and J.H.Y. analyzed data. S.L., K.I.J., H.L., Y.S.J., G.P., C.L., Y.S.O., and J.H.Y. prepared the manuscript. J. H. J., C. L., Y. S. O., and J. H. Y. edited the manuscript. All authors have read and approved the final version of the manuscript.

## FUNDING INFORMATION

This work was supported by the KBRI Basic Research Program through the Korea Brain Research Institute funded by the Ministry of Science and ICT [grant numbers 23‐BR‐02‐03, 23‐BR‐04‐02, 23‐BR‐02‐13, and 23‐BR‐05‐01]; the DGIST R&D Program of the Ministry of Science and ICT [grant number 22‐CoE‐BT‐02]; and the Small and Medium Enterprise R&D Sharing Center (SMEBridge) funded by the Ministry of Science and ICT, Republic of Korea, 2022 [Project No. A0801043001].

## CONFLICT OF INTEREST STATEMENT

The authors declare that they have no competing interests.

## Supporting information


Data S1.

File S1.

File S2.

## Data Availability

All the datasets used in this study are available from the corresponding author upon reasonable request.
