# Supplementary material for: Multi‐proteomic analyses of 5xFAD mice reveal new molecular signatures of early‐stage Alzheimer's disease
Source: Aging Cell. 2024 Mar 4;23(6):e14137. doi: 10.1111/acel.14137 (PMC11166370; doi:10.1111/acel.14137)
Supplement: Supplementary file 1 — Data S1. [file ACEL-23-e14137-s002.docx]

**Supporting information**

# Multi-proteomic analyses of 5xFAD mice reveal new molecular signatures of early-stage Alzheimer’s disease

Seulah Lee, Kuk-In Jang, Hagyeong Lee, Yeon Suk Jo, Dayoung Kwon, Geuna Park, Sungwon Bae, Yang Woo Kwon, Jin-Hyeok Jang, Yong-Seok Oh, Chany Lee, and Jong Hyuk Yoon^*^

^*^**Corresponding Author**: Jong Hyuk Yoon, Ph.D. Neurodegenerative Diseases Research Group, Korea Brain Research Institute, Daegu 41062, Republic of Korea. Tel: +82-53-980-8341; Fax: +82-53-980-8399; E-mail: [jhyoon@kbri.re.kr](mailto:jhyoon@kbri.re.kr)

**This file includes:**

**Methods**

**Supplementary Figures S1-S12**

**Supplementary Table S1**

**Supplementary Files 1-2**

**Methods**

1. **Dot blot assay**

In dot blot analysis, 2 μL of brain lysates (10 μg) were spotted onto PVDF membranes at the center of a pre-drawn grid and dried for 30 min at RT. The membranes were then blocked in TBS-T containing 5% skim milk for 30 min, washed with TBS-T, and incubated with the A11 antibody for 30 min at RT. Subsequently, the membranes were washed with TBS-T and incubated with an anti-rabbit IgG HRP-conjugated secondary antibody for 30 min at RT. Finally, the membranes were washed with TBS-T and developed using an ECL solution (ATTO Co., Ltd., Tokyo, Japan).

1. **EV preparation from human plasma**

Blood plasma was used to prepare plasma EVs following the manufacturer’s instructions. Briefly, 200 μL of plasma was incubated with 50 μL of ExoQuick exosome precipitation solution (System Biosciences, Palo Alto, CA, USA) for 30 min at 4 ℃ and centrifuged at 1,500 × *g* for 30 min at 4 ℃. The obtained EV pellet was resuspended in 50 μL of 1× DPBS (Cytiva, Marlborough, MA, USA). The isolated EVs were used to obtain proteins.


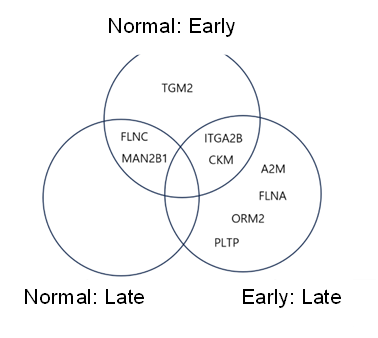


**Figure S1. Venn diagram of proteins included in the common intersection among all classes**


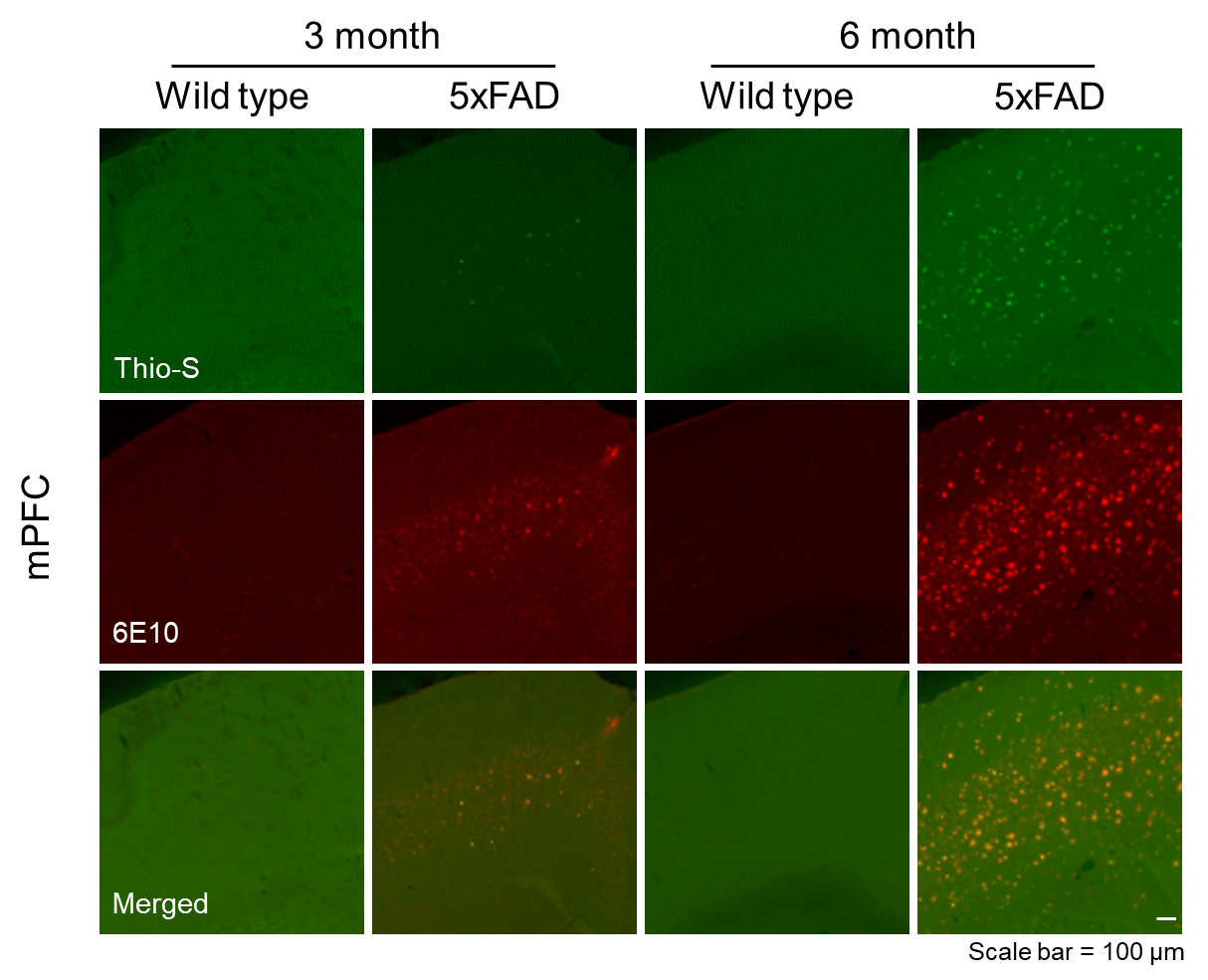


**Figure S2. Immunostained images in the medial prefrontal cortex of 5xFAD mice and age-matched wild type mice.**


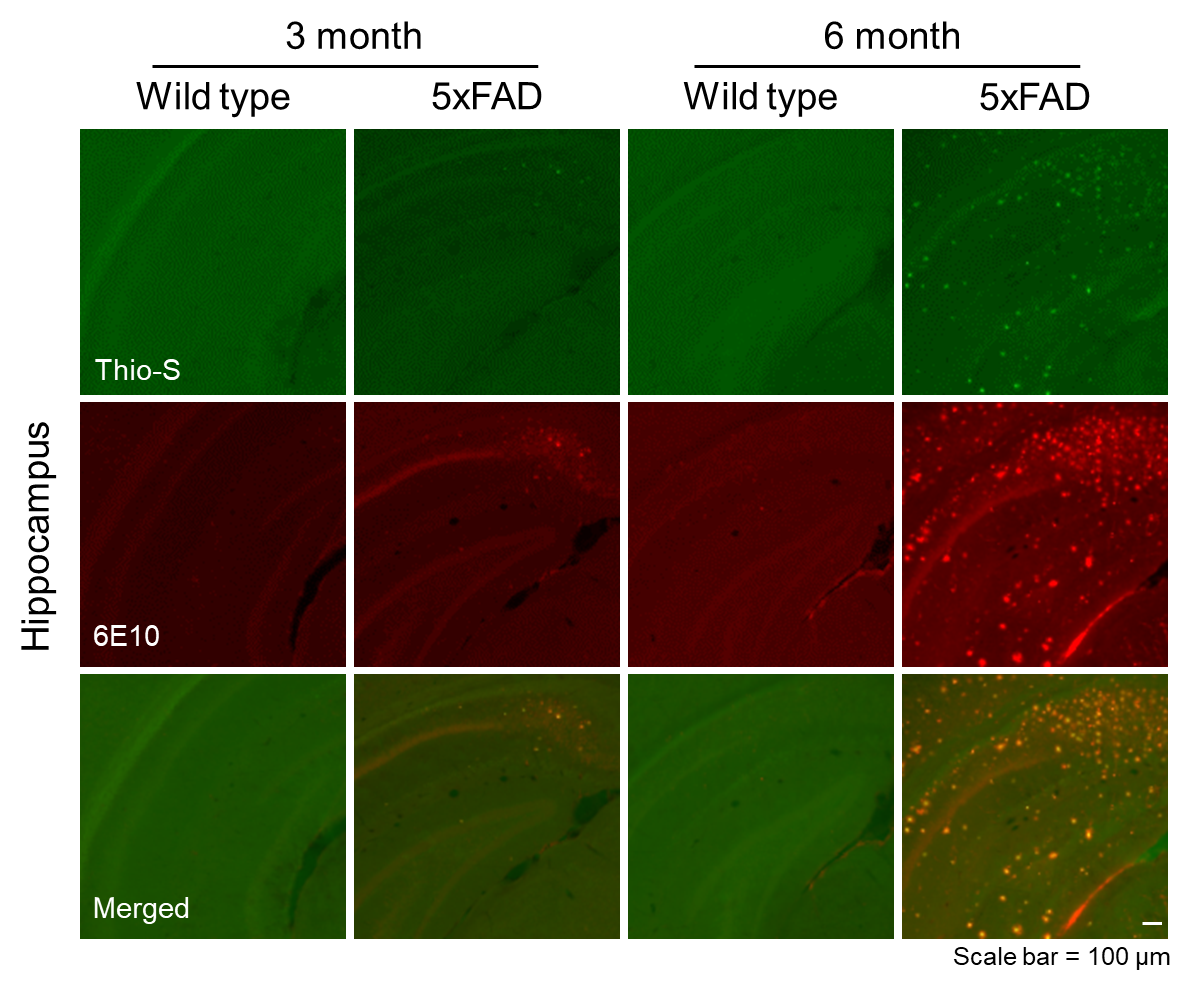


**Figure S3. Immunostained images of the hippocampus of 5xFAD mice and age-matched wild type mice.**


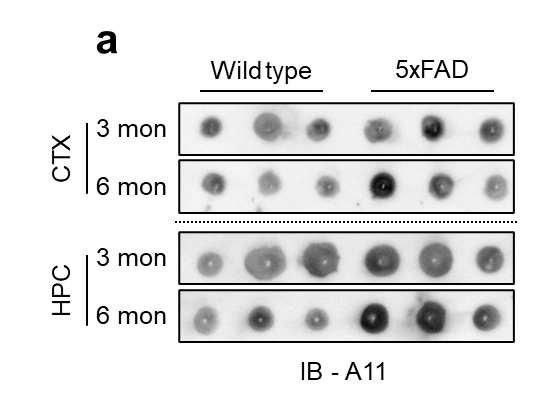


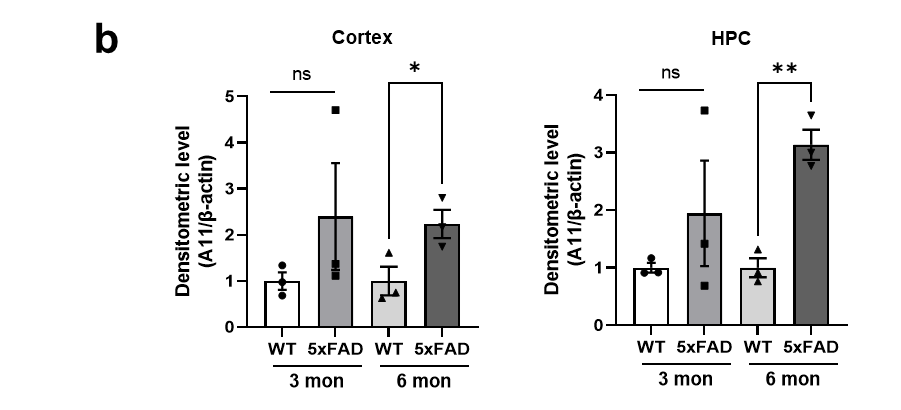


**Figure S4. Dot blot analysis using the Aβ oligomer-specific antibody A11 in 5xFAD mice.** Dot blot results were densitometrically analyzed using ImageJ and normalized by the density of β-actin. ns: not significant. ***p* < 0.01, **p* < 0.03 in two-tailed *t*-test (n = 3/group).

**
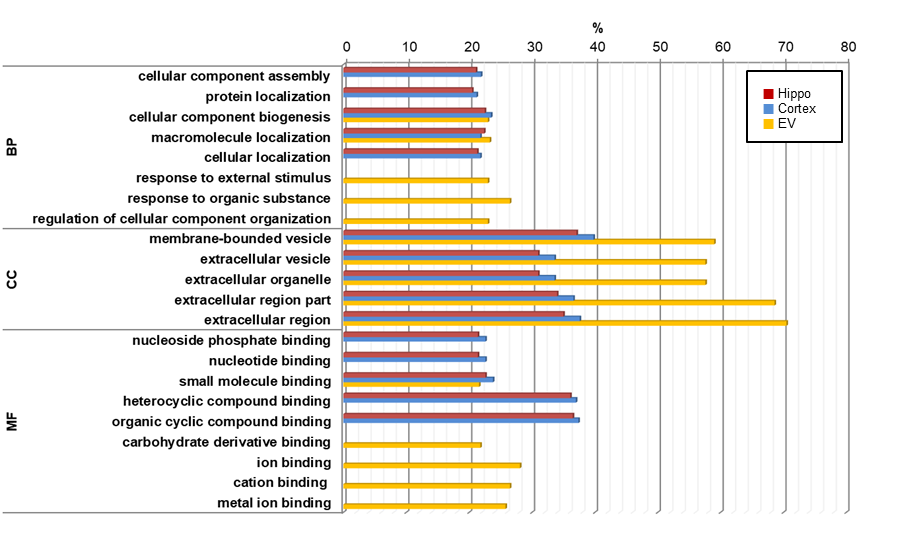
**

**Figure S5. Functional annotation of proteomic comparison between 3- and 6-month-old 5xFAD mice.** Enrichment analysis of Gene Ontology-based functional annotation among the hippocampus, cortex, and plasma extracellular vesicles. BP: biological process; CC: cellular component; MF: molecular function.


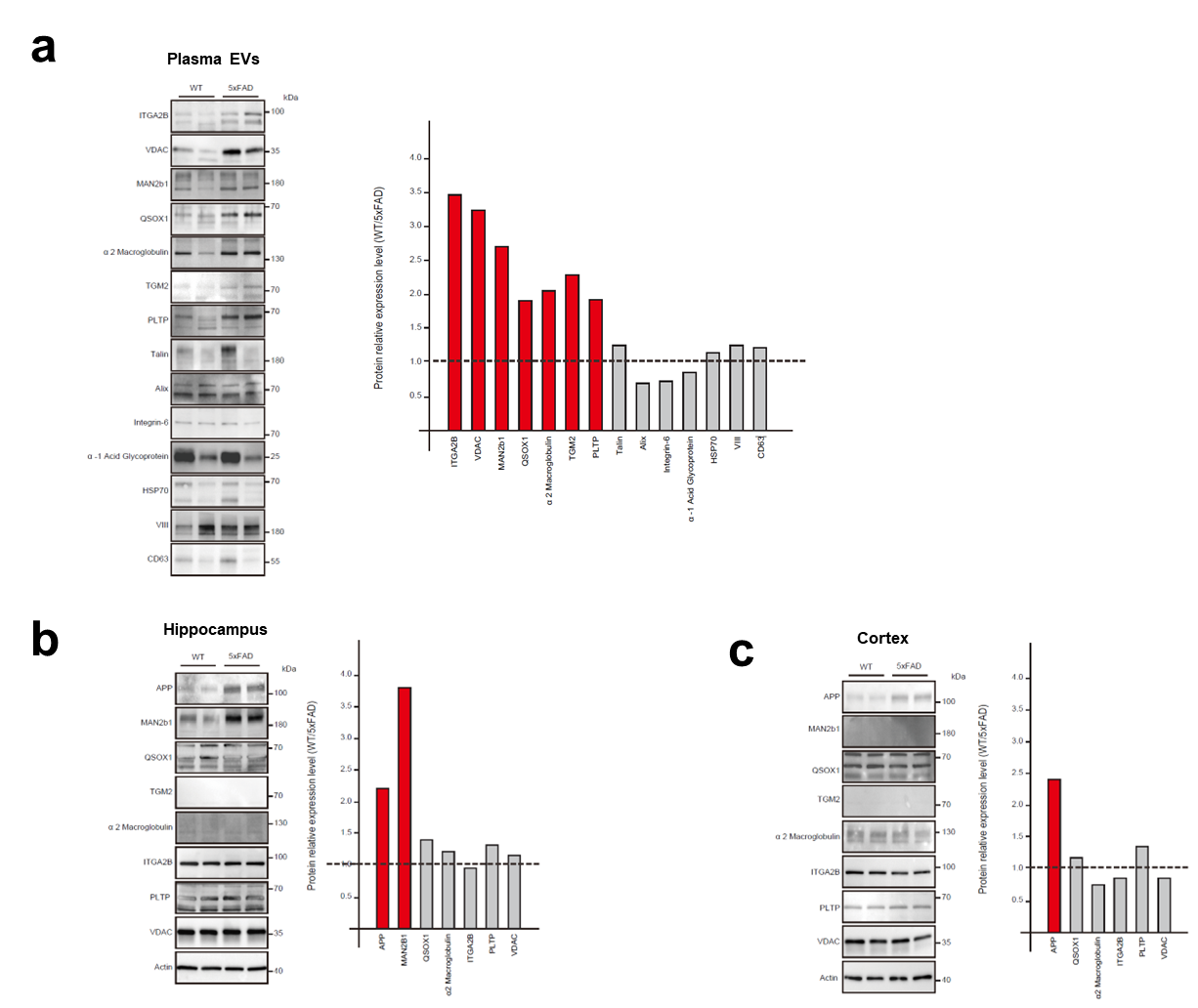


**Figure S6.** **Western blotting of selected biomarker candidates in 5xFAD mice.** Western blotting of selected biomarker candidates in the plasma EVs, hippocampus, and cortex of 3-month-old WT and 5xFAD mice. The selected biomarker candidates were identified from comparative proteomics results. a, Western blotting of selected biomarker candidates using plasma EVs; CD63 is a molecular marker of EVs. Western blotting of selected biomarker candidates in the b, hippocampus and c, cortex. Graphs on the right side of each panel denote densitometric analysis of western blotting results performed using ImageJ Ver. 1.53. The red bar indicates a statistically significant fold change (p < 0.05).


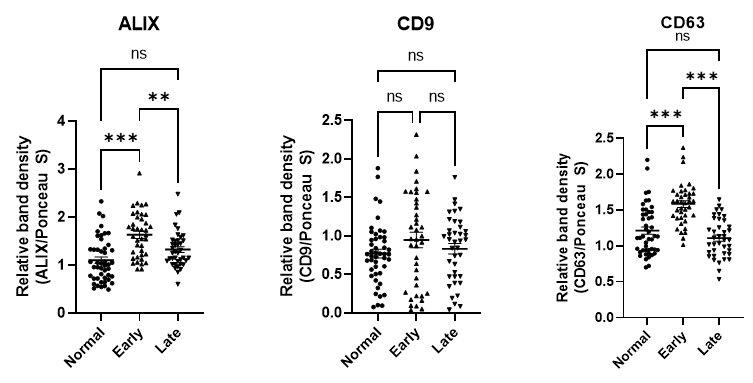


**Figure S7. Western blots of the extracellular vesicle (EV) markers using blood EVs of stage-divided patients with Alzheimer’s disease (AD).** Western blots of the EV markers in three different human plasma EVs, namely, healthy, early-stage patients with AD, and late-stage patients with AD. Plasma EVs were blotted using the corresponding antibodies. A scatter plot of marker proteins, namely, ALIX, CD9, and CD63 is shown. Western blot results were densitometrically analyzed using ImageJ and normalized by the density of bands stained with Ponceau S. ns: not significant. ****p* < 0.001, ***p* < 0.01, **p* < 0.03 in one-way ANOVA with Bonfferoni’s multiple comparisons (n = 39–47/group).


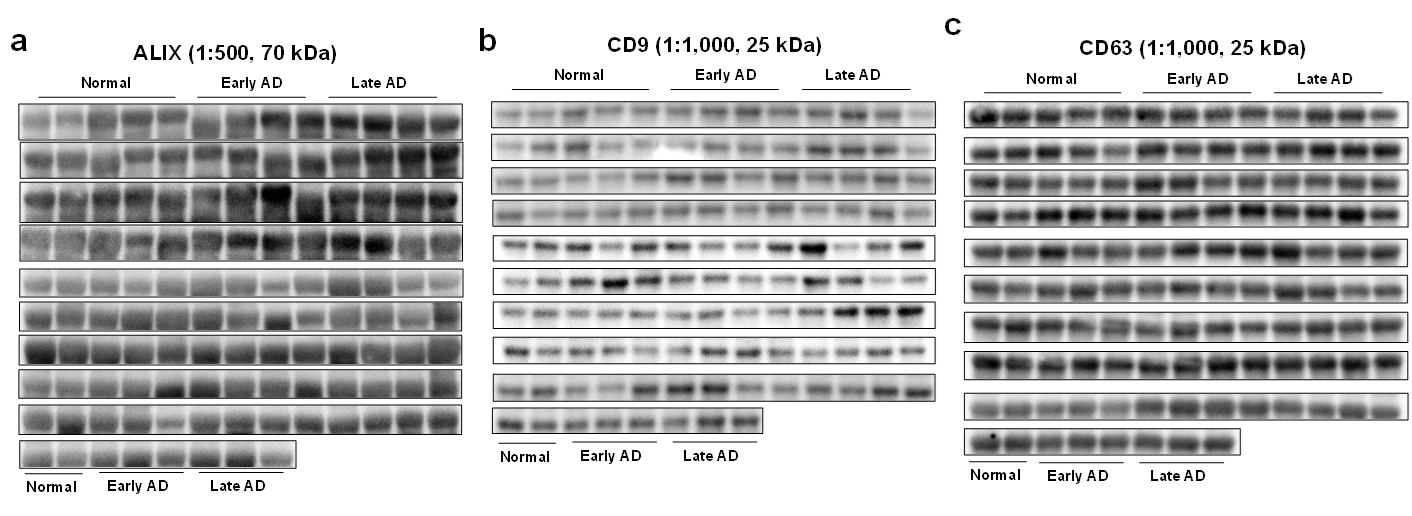


**Figure S8. Western blots of the EV markers using blood EVs of stage-divided patients with AD.** Western blot of the EV markers in three different human plasma EVs, namely, healthy, early-stage patients with AD, and late-stage patients with AD.


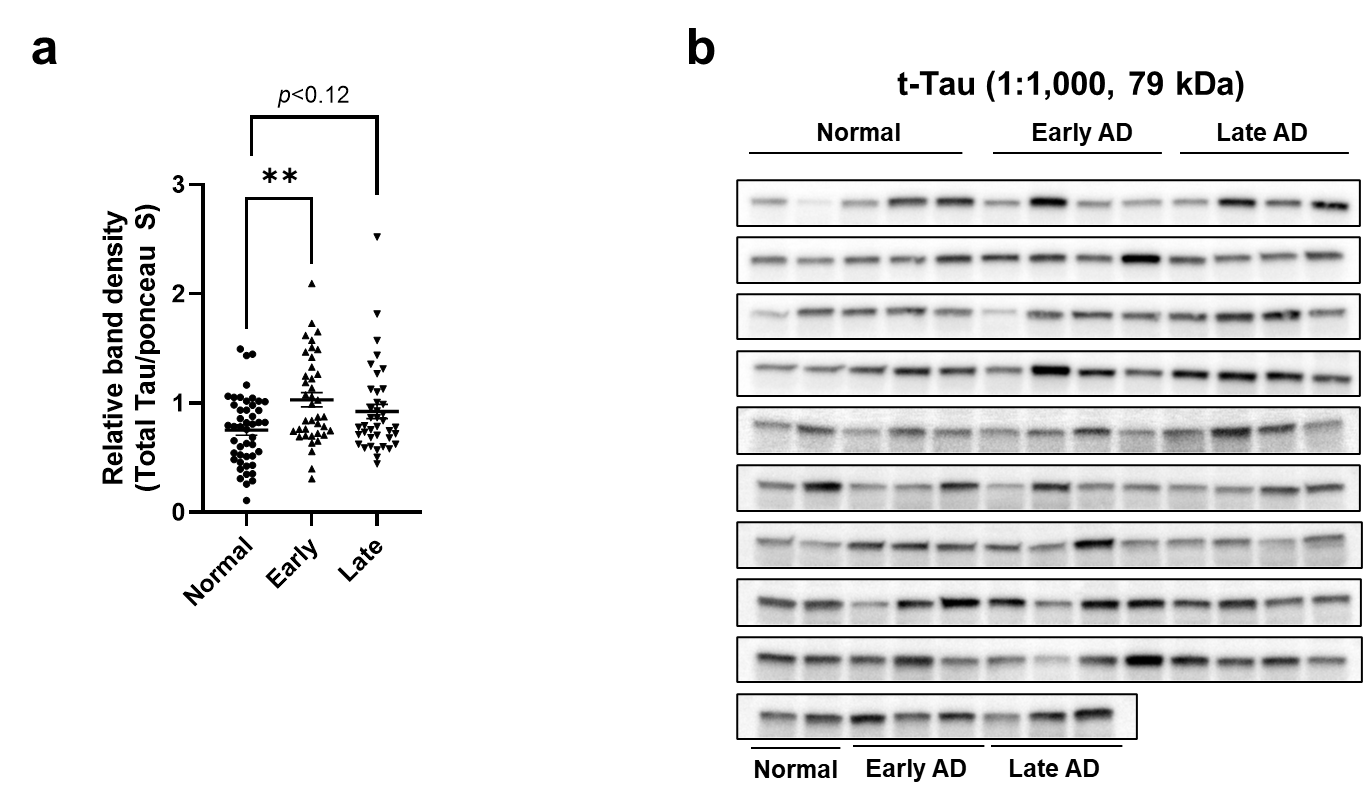


**Figure S9. Western blots of the Tau protein validation using plasma EVs of stage-divided patients with AD.** Western blot of Tau protein in three different human plasma EVs, namely healthy, early-stage patients with AD, and late-stage patients with AD. Plasma EVs were blotted using the corresponding antibodies. A scatter plot of Tau protein is shown. Western blot results were densitometrically analyzed using ImageJ and normalized by the density of bands stained with Ponceau S. ***p* < 0.01 in one-way ANOVA with Bonfferoni’s multiple comparisons (n = 39–47/group).


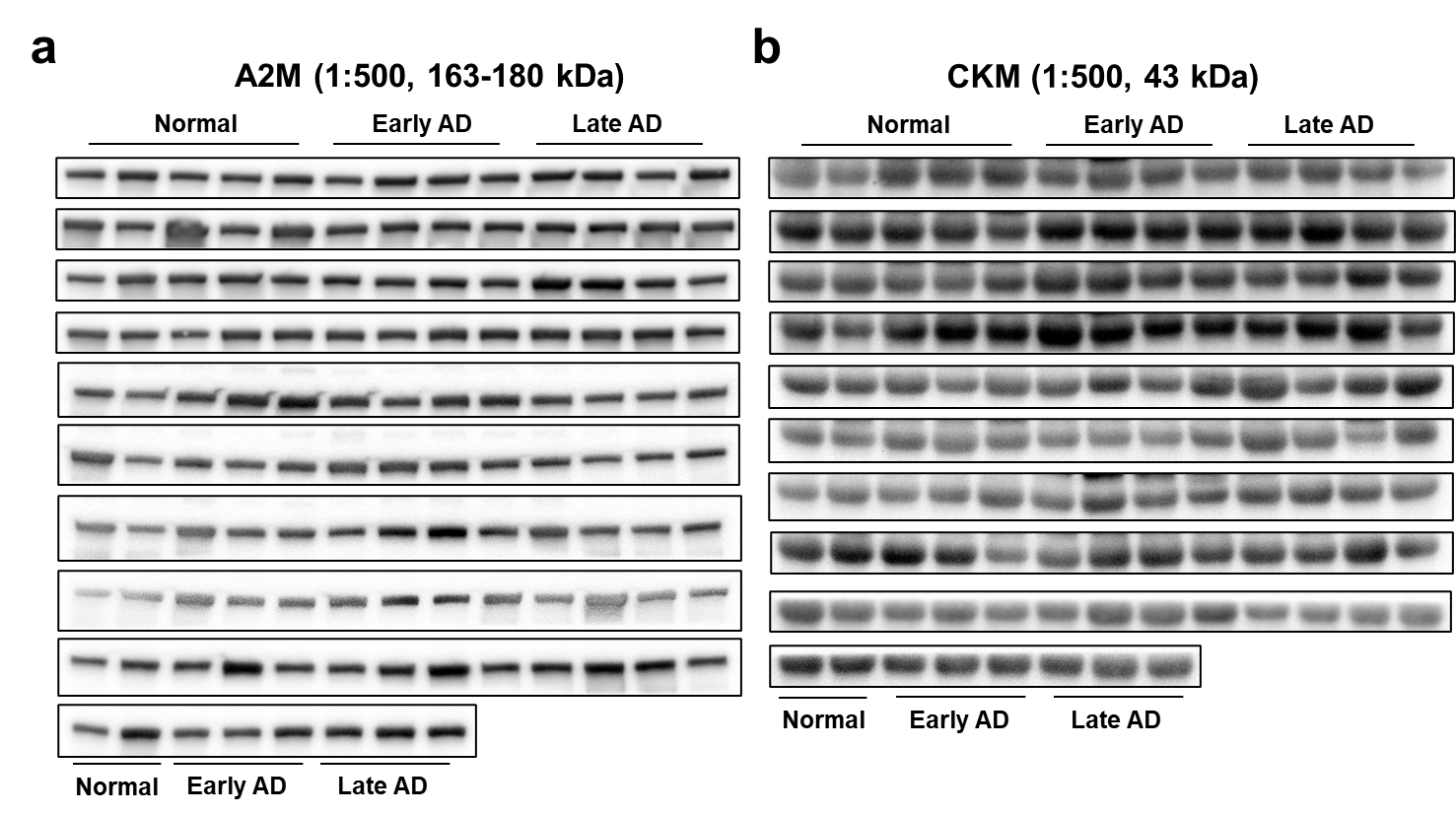


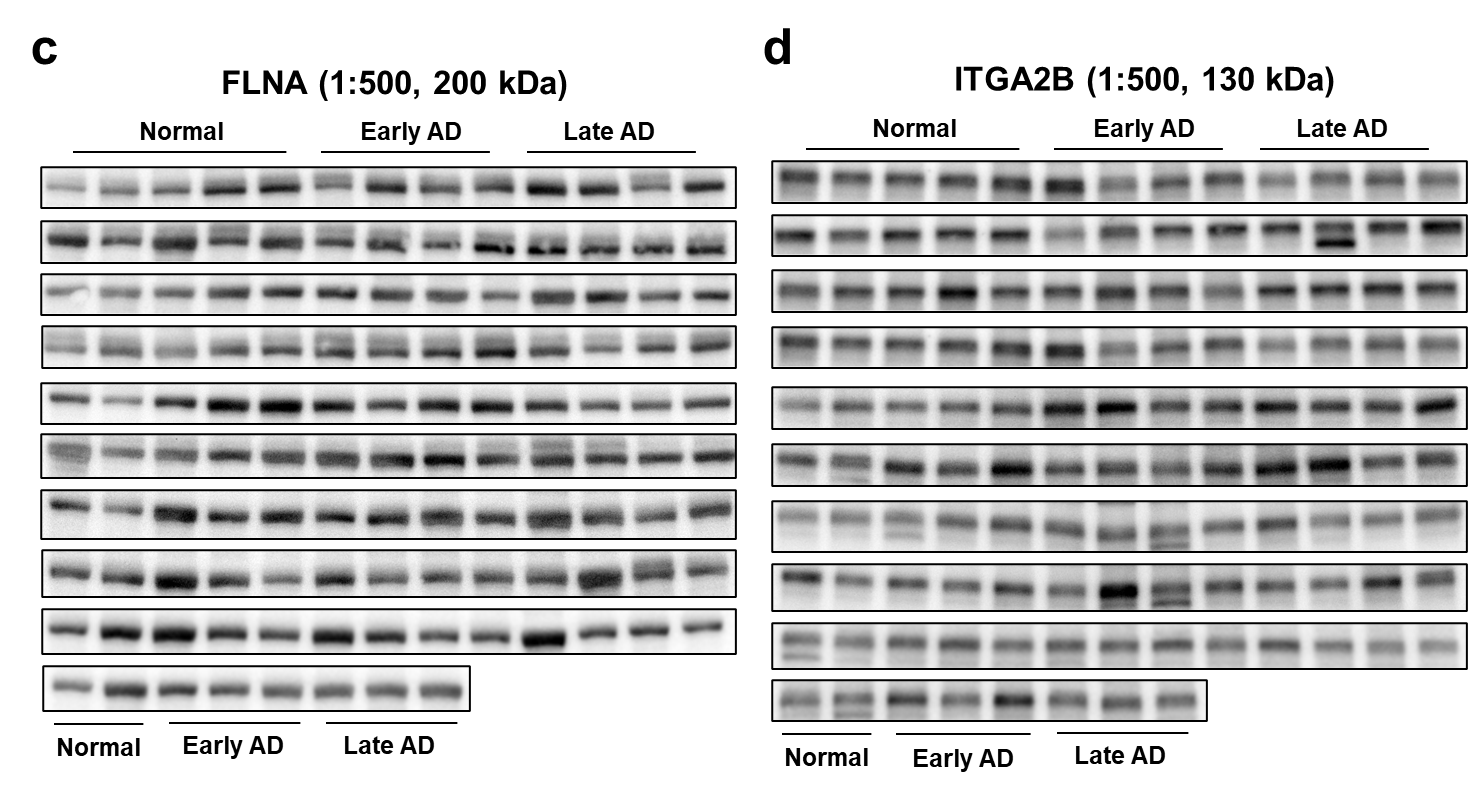


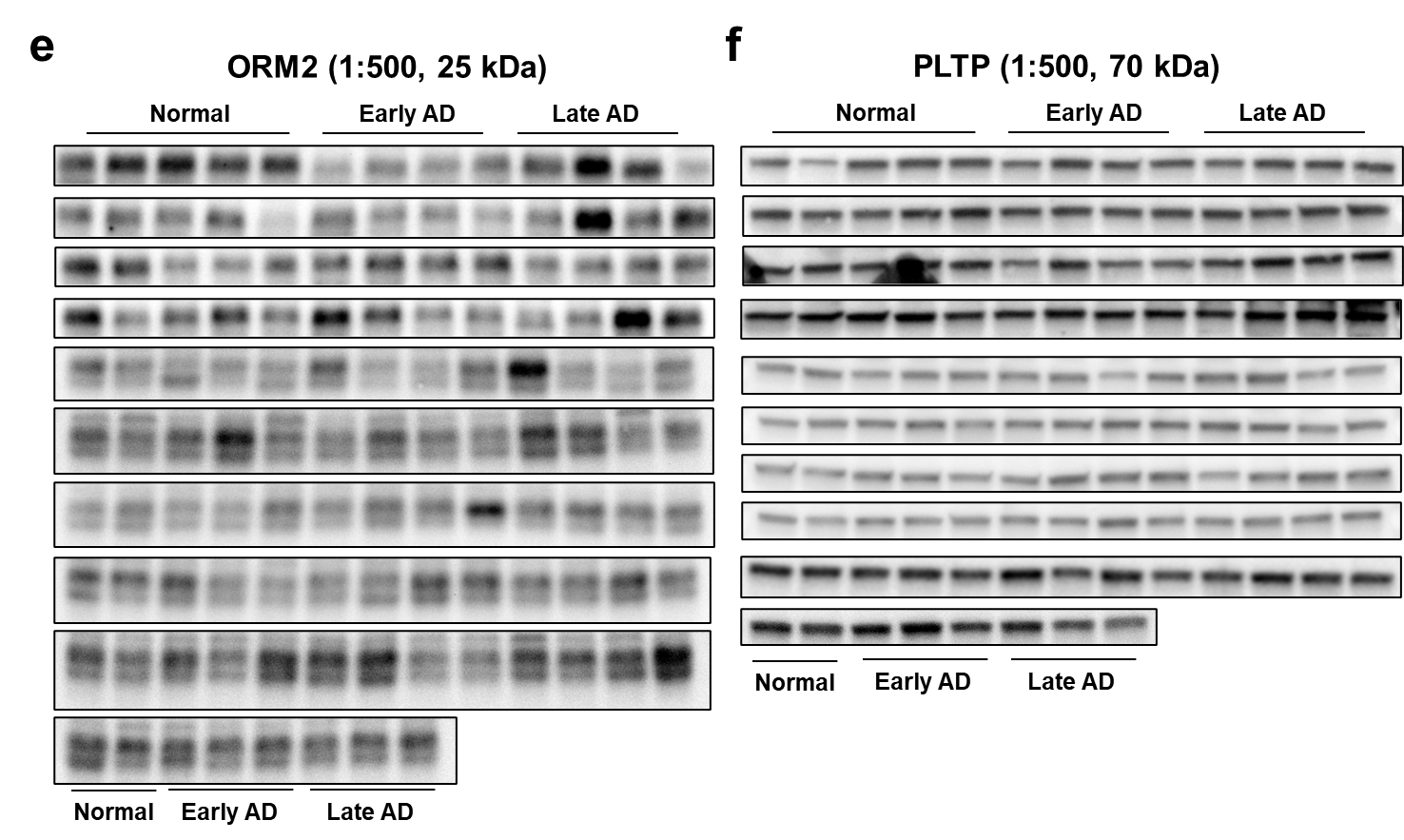


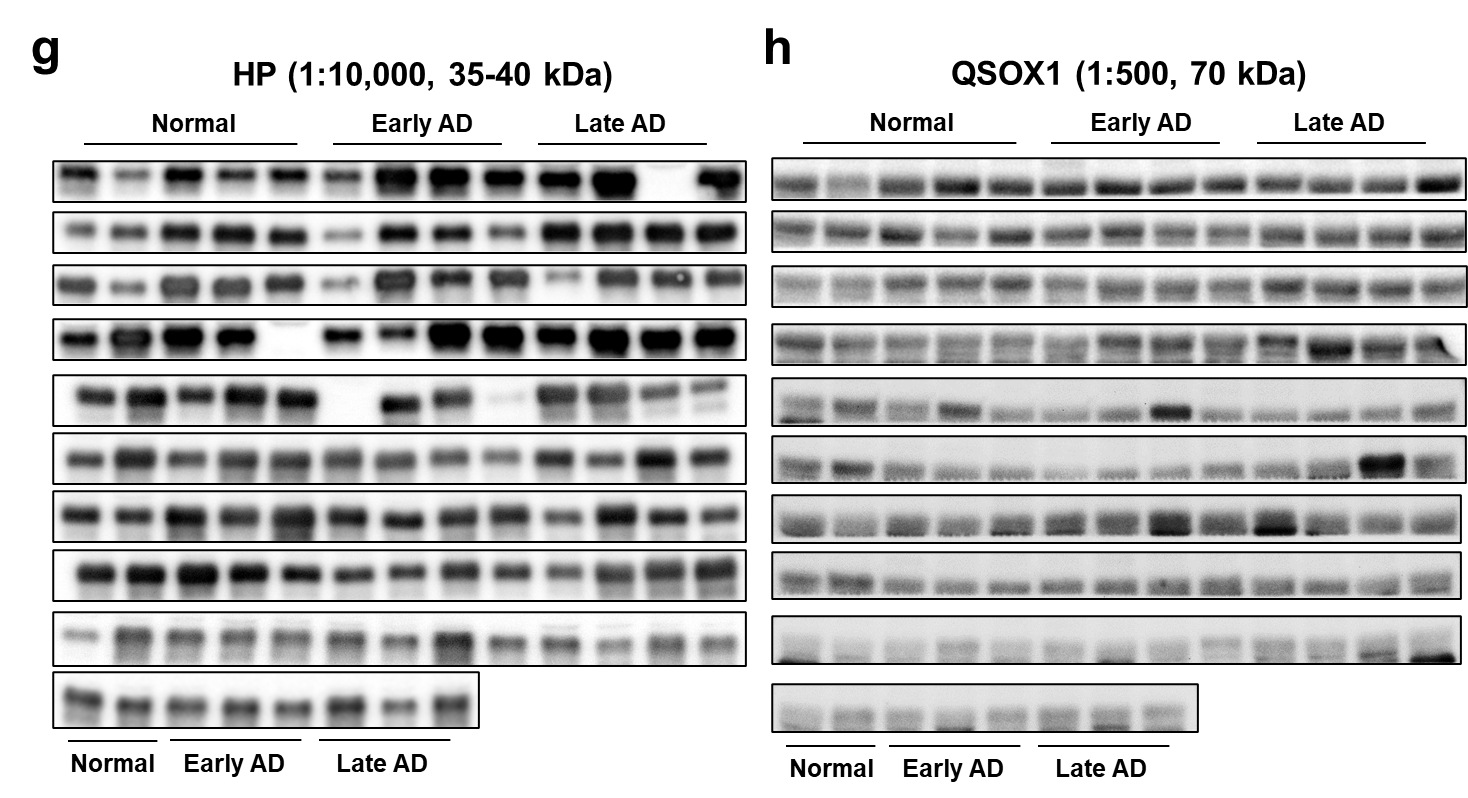


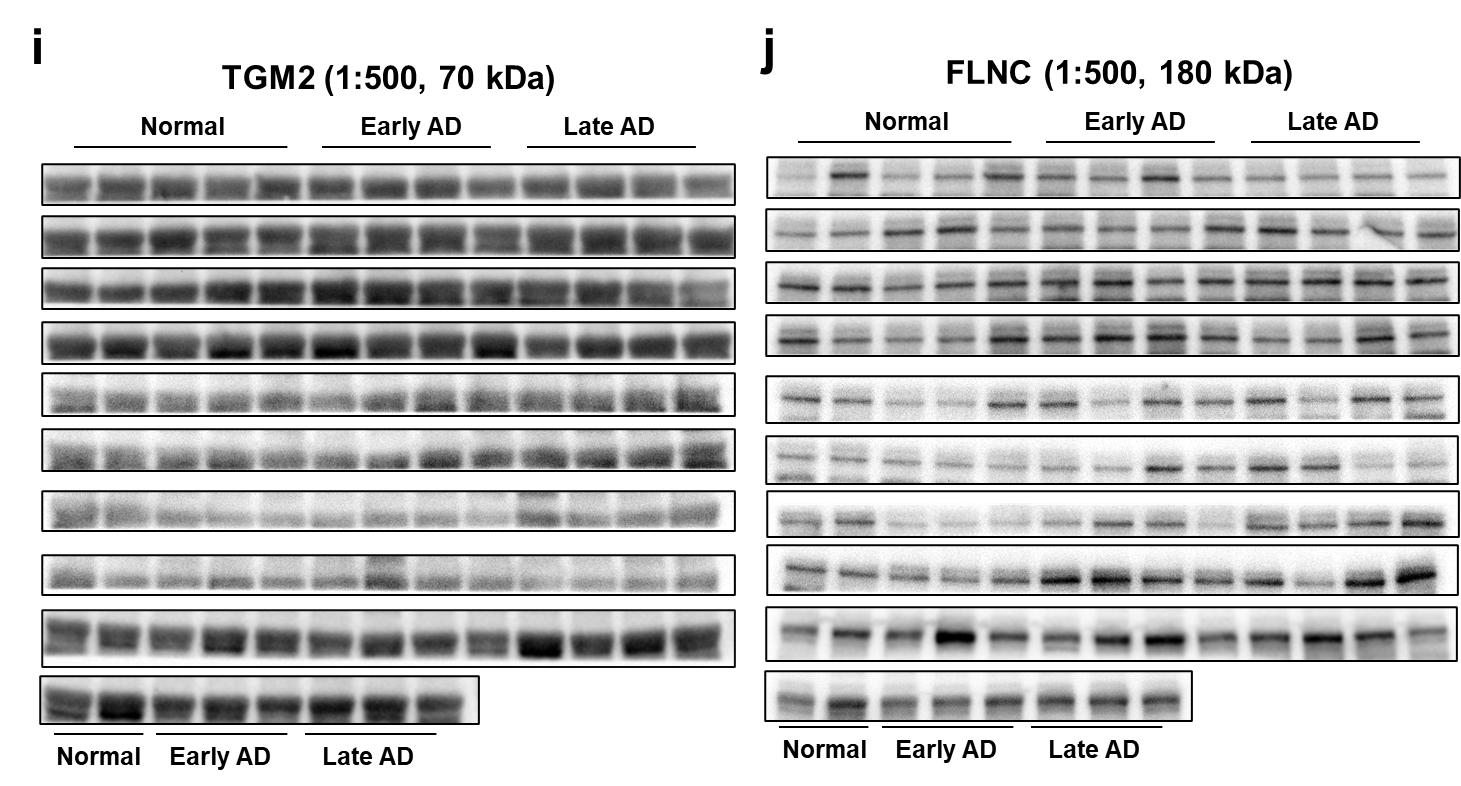


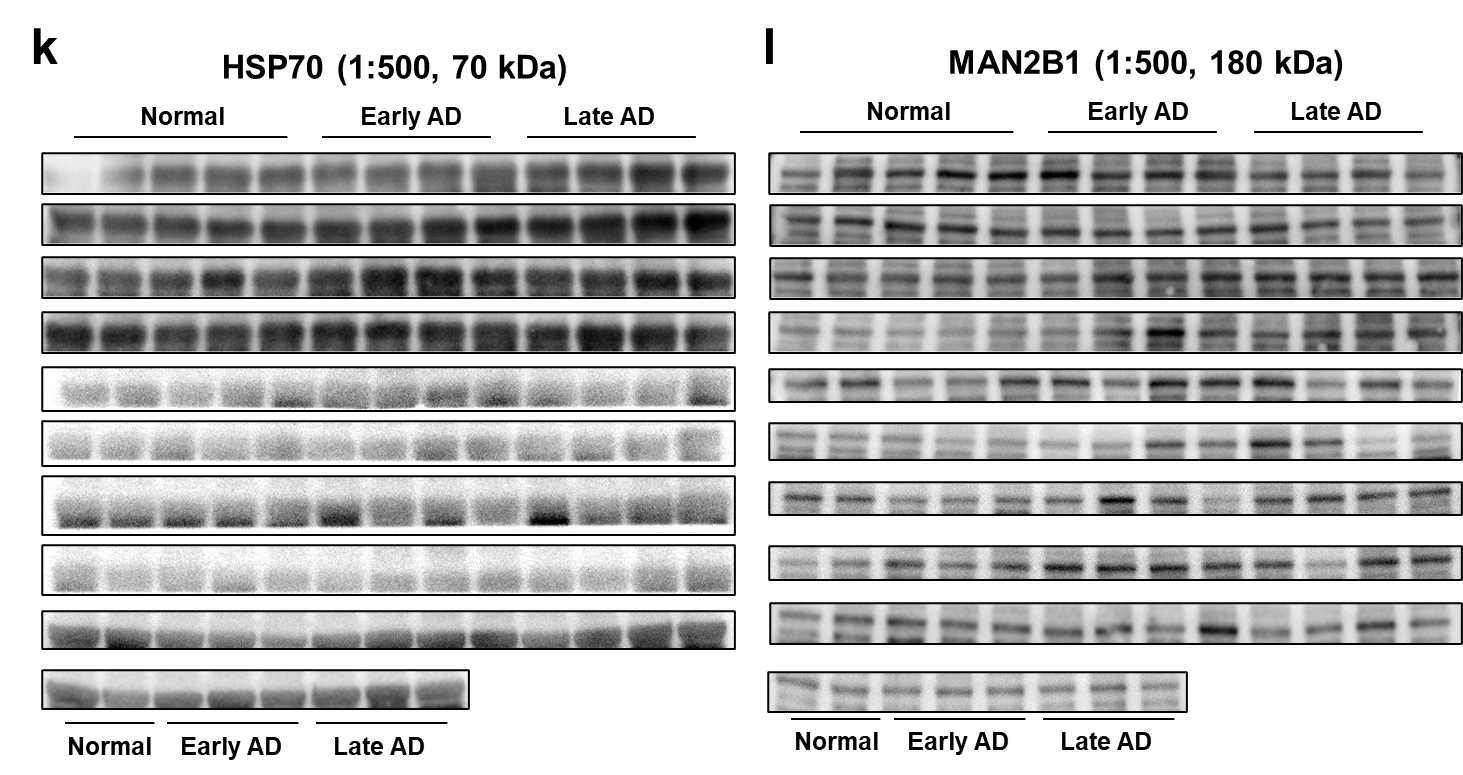


**Figure S10. Western blots of selected biomarker candidates using blood EVs of stage-divided patients with AD.** Western blot of the selected biomarker candidates in three different human plasma EVs, namely, healthy, early-stage patients with AD, and late-stage patients with AD.


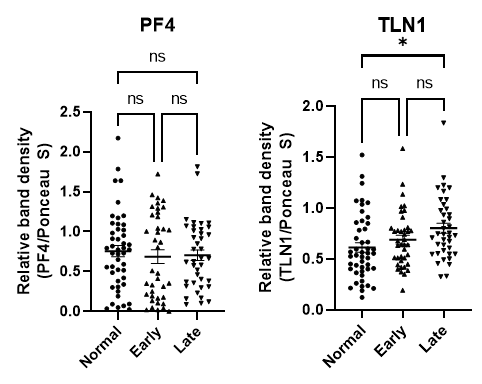


**Figure S11. Western blots of selected biomarker candidates using plasma EVs of stage-divided patients with AD.** Western blot of the selected biomarker candidates in three different human plasma EVs, namely, healthy, early-stage patients with AD, and late-stage patients with AD. Plasma EVs were blotted using the corresponding antibodies. A scatter plot of biomarker candidate proteins, including PF4 and TLN1, is shown. Western blot results were densitometrically analyzed using ImageJ and normalized by the density of the band stained with Ponceau S. ns: not significant.


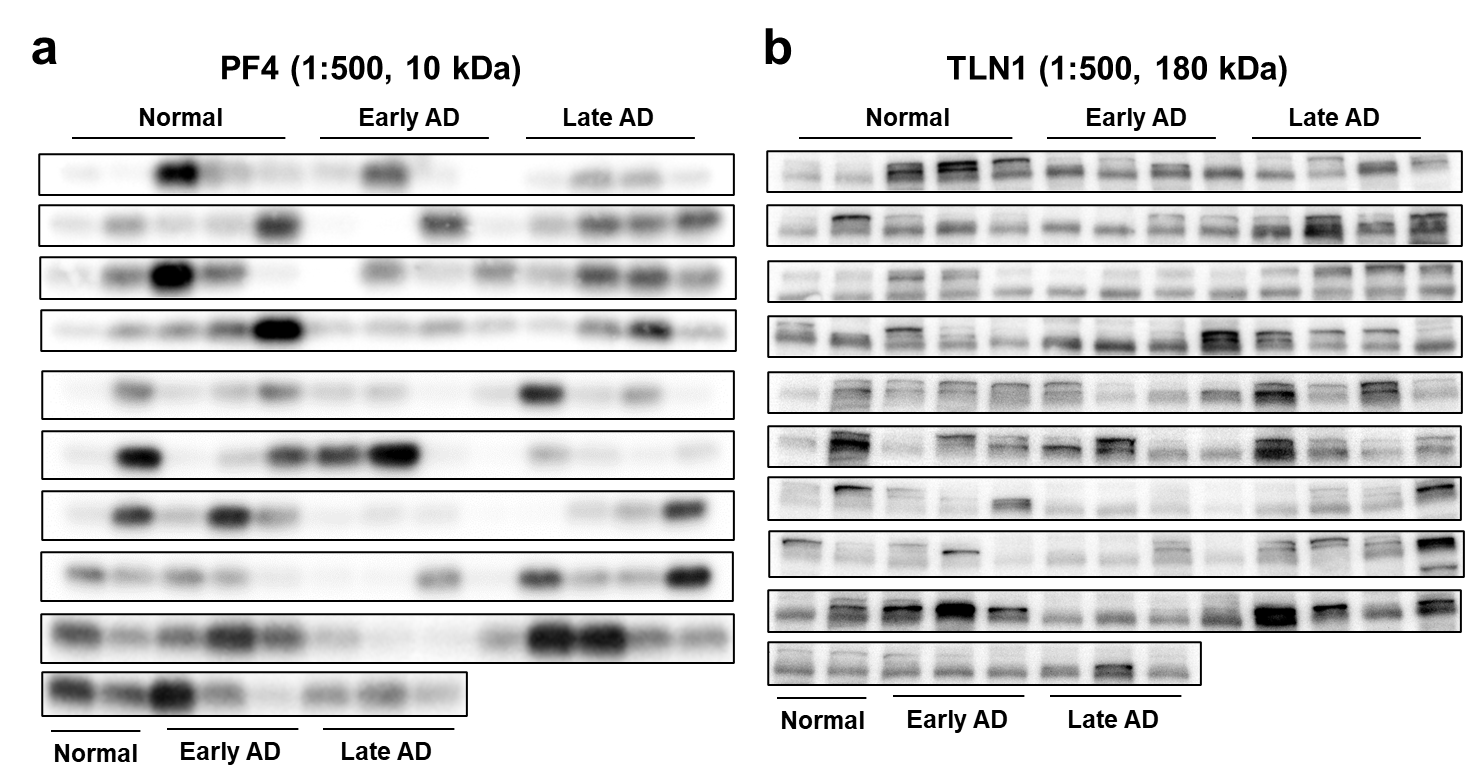


**Figure S12. Western blots of non-significant biomarker candidates using blood EVs of stage-divided patients with AD.** Western blot of the selected biomarker candidates in three different human plasma EVs, namely, healthy, early-stage patients with AD, and late-stage patients with AD.

**Supplementary Table S1. List of antibodies used in immunoblotting**

| Target name | Cat no. | Host | Dilution titer | Company |
| --- | --- | --- | --- | --- |
| A11 (Aβ oligomer) | AB9234 | Rabbit | 1:1,000 | Millipore |
| ALG-2-interacting protein X (ALIX) | ab275377 | Rabbit | 1:500 | Abcam |
| Alpha-2-macroglobin (A2M) | ab58703 | Rabbit | 1:500 | Abcam |
| Phospho protein kinase B (Akt) | 9271S | Rabbit | 1:1,000 | Cell Signaling Technology |
| Total Akt | 9272S | Rabbit | 1:1,000 | Cell Signaling Technology |
| Amyloid precursor protein (APP) | MAB348 | Mouse | 1:1,000 | Sigma-Aldrich |
| Cluster of differentiation 63 (CD63) | sc-5275 | Mouse | 1:1,000 | Santa Cruz Biotechnology |
| CD9 | MA5-31980 | Rabbit | 1:1,000 | Invitrogen |
| Creatine kinase M-type (CKM) | ab233201 | Rabbit | 1:500 | Abcam |
| Filamin-A (FLNA) | LS-B7557-50 | Rabbit | 1:500 | LS Bio |
| FLNC | ARP64454_P050 | Rabbit | 1:500 | AVIVA System |
| Haptoglobin (Hp) | ab256454 | Rabbit | 1:10,000 | Abcam |
| Heat shock protein 70 (HSP70) | MA3-006 | Mouse | 1:500 | Invitrogen |
| Integrin alpha-IIb (ITGA2B) | MBS2521429 | Rabbit | 1:500 | MyBioSource |
| Lysosomal alpha-mannosidase (MAN2B1) | MBS9130534 | Rabbit | 1:500 | MyBioSource |
| Orosomucoid 2 (ORM2) | ab231906 | Rabbit | 1:500 | Abcam |
| Platelet factor 4 (PF4) | ab129183 | Rabbit | 1:500 | Abcam |
| Phospholipid transfer protein (PLTP) | LS-B295 | Rabbit | 1:500 | LS Bio |
| Isoform 3 of quiescin sulfhydryl oxidase 1 (QSOX1) | 12713-1-AP | Rabbit | 1:500 | Proteintech |
| Total Tau | ab64193 | Rabbit | 1:1000 | Abcam |
| Protein-glutamine gamma-glutamyltransferase 2 (TGM2) | PA5-29357 | Rabbit | 1:500 | Invitrogen |
| Talin 1 (TLN1) | ab108480 | Mouse | 1:500 | Abcam |
| β-Actin | Sc-47778 | Mouse | 1:1000 | Santa Cruz Biotechnology |
| β-Amyloid 1-16 | SIG-39320 | Mouse | 1:1000 | BioLegend |

**Supplementary File 1. List of proteins identified using proteomic analysis**

**Supplementary File 2. Patient information**
